# Optical and Computational Studies on a Triazine Derivative of Dual Fluorescence Enhancement/Superquenching Upon Nanoparticle Interactions

**DOI:** 10.1007/s10895-025-04474-w

**Published:** 2025-09-25

**Authors:** Khloud M. Elhalby, Ahmed H. Mangood, Mahmoud A. S. Sakr, Ahmed A. El-Barbary, El-Zeiny M. Ebeid, Heba A. El-Shekheby

**Affiliations:** 1 Chemistry Department, Faculty of Science, Menoufia University, Shebin El-Kom, Egypt; 2 Chemistry Department, Center of Basic Science, Misr University for Science and Technology (MUST), P.O.77, Giza, Egypt; 3 Chemistry Department, Faculty of Science, Tanta University, Tanta, 31527 Egypt

**Keywords:** 1,2,4-Triazine, Photophysical properties, Optical properties, NPs characterization, Superquenching, DFT calculations

## Abstract

Derivatives of 1,2,4-triazine have emerged as versatile scaffolds with broad pharmacological and biological potential, prompting deeper exploration of their optical behavior. In this study, we investigate the photophysical properties of a novel 1,2,4-triazine dye, 4-amino-6-(4-methoxyphenyl)-1,4,6,7 tetrahydrothieno [2,3-e] [1,2,4] triazine-3(2 H)-thione (Triazine I) with a focus on its interaction with noble metal nanoparticles. The synthesized dye was structurally characterized using FTIR, ^1^H-NMR, ^13^C-NMR, and mass spectrometry to confirm its purity and molecular structure. Silver (Ag-NPs) and gold (Au-NPs) nanoparticles were synthesized via a chemical reduction method and thoroughly characterized using UV–vis spectroscopy, TEM, DLS, Zeta Potential, and XRD, confirming their successful formation, stability, and crystalline nature. Spectroscopic investigations revealed solvent-dependent absorption and emission behavior of Triazine I, along with a dual fluorescence response in the presence of nanoparticles: superquenching by Ag-NPs via non-radiative decay enhancement, and fluorescence amplification by Au-NPs through radiative enhancement (MEF). These findings open promising avenues for using such systems in advanced fluorescence-based biosensing and immunoassays. Furthermore, DFT-based computational studies provided insights into molecular geometry, electronic transitions, and electrostatic potential, reinforcing experimental observations and guiding future molecular de sign. This integrated experimental–theoretical approach highlights Triazine I as a promising candidate for tunable optical applications, particularly in bioimaging and diagnostic platforms.

**Supplementary Information:**

The online version contains supplementary material available at 10.1007/s10895-025-04474-w.

## Introduction

**Fig. 1:**
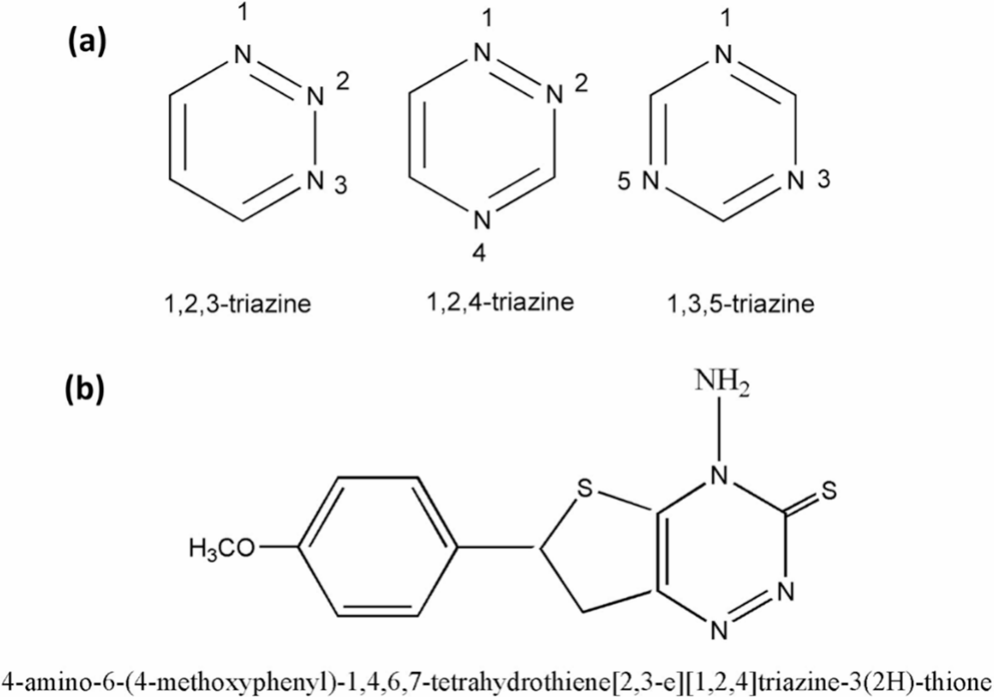
Structures of (**a**) triazine isomers according to the location of the nitrogen atoms and (**b**) 1,2,4-triazine dye: 4-amino-6-(4-methoxyphenyl)−1,4,6,7 tetrahydrothiene [2,3e] [1, 2, 4] triazine-3(2 H)-thione (Triazine I)

Heterocycle chemistry has garnered considerable attention recently due to its diverse pharmaceutical and biological applications [1]. Triazines are an important class of heterocyclic compounds with the empirical parent formula C_3_H_3_N_3_, which Scheele first synthesized through pyrolysis of uric acid in 1776. The triazine structure is a six-membered, heterocyclic benzene ring with nitrogen substituting for three carbons. The names 1, 2, 3-triazine, 1, 2, 4-triazine, and 1, 3, 5-triazine refer to the isomers of triazine, which are distinguished from each other based on the locations of their nitrogen atoms according to Fig. 1(a). Triazine derivatives are used as pesticides, dyestuffs, optical bleaches, explosives, surface-active agents, and in the textile industry [2].

The biological and pharmacological activities of triazine isomers and their derivatives are recognized for having significant uses, such as PD-L1 inhibitors [3], strong effect on AR-dependent 22Rv1 prostate cancer cells in an in vitro assay and antiproliferative activity, particularly against the MCF7 cell line, with GI50 values in the micromolar range [4]. Altretamine, gedatolisib, and enasidenib are three s-triazine compounds that have already received approval for the treatment of leukemia, metastatic breast cancer, and refractory ovarian cancer. Many protein kinases are the subject of current research on s-triazine compounds that show tremendous potential for antitumor activity [5]. Also, triazine derivatives have a vital impact on the design and development of anticancer drugs. They have been extensively employed as donors in bulk hetero junction solar cells, thermally active delayed fluorescence emitters (TADFs), and electron transport in organic light emitting diodes (OLEDs) [6].

1,2,4-triazine and their analogs exhibit a broad range of numerous natural, synthetic biologically and pharmacologically active targets, mainly antimicrobial, antiproliferative properties [7], antimalarial [8], antifungal [9], antibacterial [10], anticancer [11], anti-HIV and antiHBV [12], antioxidant, anti-inflammatory [13], antidepressant [14], and therapeutic [15]. The kind of nucleophile applied, the stability of transition state adducts, site selectivity, and the solvent’s dielectric constants all affect the chemical reactivity of 1,2,4 triazines. In addition to their significant biological and pharmacological applications, 1,2,4-triazine’s photophysical characteristics have sparked great fascination. The photophysical characteristics of this category of heterocyclic compounds result from a high level of pi-conjugated charge transfer and the donor-acceptor interaction.

Because of the numerous application domains of metallic nanoparticles, studying the interaction between fluorophores of organic heterocyclic molecules and these nanoparticles has been regarded as a crucial aspect of nanotechnology research. When a dye molecule is close to metallic nanoparticles, its fluorescence is either increased or quenched. These phenomena can be utilized for molecular imaging or sensing applications [16]. A dye molecule’s emission behavior can be altered by metallic nanoparticles, like silver and gold, that have a visible wavelength range plasmonic resonance.

Studying the fluorescence quenching of organic heterocyclic molecules has emerged as a crucial spectroscopic methodology for studying biochemical and biophysical processes [17]. Fluorescence intensity quenching can be caused by several methods. The two most common methods are static or instantaneous quenching, in which the dye molecules are adsorbed on metallic nanoparticles’ surface, causing a non-luminous complex to develop between the quencher and fluorophore, resulting in an overall decrease in emission intensity. Another quenching mechanism is dynamic or collisional quenching, which involves an excited fluorophore’s direct collision or interaction with a quencher during its lifetime in its excited state. For quenching to arise dynamically or statically, contact between the quencher and the fluorophore is required. Moreover, quenching may occur through several methods, including energy transfer, intersystem crossing, excited state reactions, molecular rearrangements, and charge transfer complexes that form in both the ground and excited states [18]. Important factors influencing the quenching process include the concentration of the quencher, the solvent medium’s polarity, and the characteristics of the quencher molecules and fluorophore. The term “super-quenching” or “hyper-quenching” has been used when the quenching constant for Stern-Volmer (K_SV_ ~ 10^7^− 10^10^ mol^−1^ dm^3^) becomes multiple orders of magnitude in comparison to the normal processes that quench fluorescence (~ 10^2^ mol^−1^dm^3^). Super-quenching by nanoparticles has uses in optical materials, biosensing, and scanning probe microscopy [19].

One of the significant applications of localized surface plasmons is the fluorescence enhancement of the dye. Gersten and Nitzan have theoretically investigated fluorescence resulting from the dye molecule adsorbed on a spherical metal particle. They noted that the transfer of energy from the excited dye to the particle of metal and the enhancement of a local field compete with each other. When metallic nanoparticles are present, the enhancement process occurs due to the strong interaction between the surface plasmon of these nanoparticles and the fluorophore’s electronic transition dipole moment [20]. The dye molecule’s quantum yield, the particle’s size and shape, and the distance between the surface and the dye all have notable effects on enhancing fluorescence intensity.

This research aims to synthesize and comprehensively characterize Triazine I, a fused thieno-triazine compound, as well as the prepared silver and gold nanoparticles (Ag-NPs and Au-NPs), to investigate their roles in metal-selective fluorescence modulation. The study is driven by the hypothesis that the photophysical behavior of Triazine I is highly sensitive to environmental factors such as solvent polarity and nanoparticle interactions. This study presents the first report of metal-selective dual fluorescence modulation in fused heterocyclic systems, demonstrating superquenching (“turn-off”) with Ag-NPs and significant enhancement (“turn-on”) with Au-NPs, unlike typical systems showing only single responses. By integrating experimental measurements with theoretical analyses (DFT/TD-DFT), this work provides new mechanistic insights into how thieno fusion influences electrostatic potential distribution, paving the way for future applications in optical sensing and bioimaging.

## Experimental

### Materials

Requested chemicals and solvents were analytical grade and used without extra purification. Tetrachloroauric acid (99.9%, HAuCl_4_.3H_2_O), Silver nitrate (AgNO_3_) and citrate trisodium salt (95%, C_6_H_5_O_7_Na_3_⋅2H_2_O) were obtained from Sigma-Aldrich, ethanol (EtOH), ethylene glycol (EG) and quinine sulfate were purchased from Fluka. Aqueous solutions were prepared using Ethanol.

### Instruments and Characterization Techniques

A Shimadzu-50 UV-vis. the spectrophotometer was used to measure the absorbance spectra of the samples and the prepared Ag/Au nanoparticles. The JASCO FP-8200 spectrofluorometer was used to record the fluorescence spectra using a rectangular quartz cuvette (1 × 1 × 4.5 cm³) using Xe as the origin of light. FT-IR spectra were obtained in the range of 4000 to 400 cm^−1^ using a JASCO FT/IR-4100 spectrophotometer. NMR spectra, both ^1^H and ^13^C, were acquired with a Bruker AC spectrometer (400 MHz) using DMSO as a solvent. Mass spectra were measured with a Finnigan MAT 8222 EX mass spectrometer at 70 eV. TEM measurements of silver and gold nanoparticles were examined at 200 kV using HRTEM (JEM 2100), and gold nanoparticles were measured using a JEM-100SX model electron microscope. Samples were loaded on carbon-coated Cu grids (200 mesh). The average particle size, size distribution of particles, and Zeta potential were observed in the nano silver and nano gold fluids by (Brookhaven, USA) Particle Size - Zeta Potential Analyzer. This instrument is capable of measuring particle size by illuminating the particles with a laser of wavelength 658 nm (He–Ne), a scattering angle of 90°, a measurement temperature of 25 °C, and a medium viscosity of 0.892 mPa·s. XRD analysis was conducted at 35 kV and 25 mA using a Shimadzu diffractometer with monochromatic Cu Kα radiation (*λ* = 0.154 Å). The scan rate was set to 2° min⁻¹, covering a 2θ range from 30° to 80°. Powder samples for the analysis were prepared from nanoparticle-containing solutions by freezing and drying at 80 °C. The picosecond fluorescence decay profiles were measured by the time-correlated single photon counting (TCSPC) method using FluoroHub (Horiba Scientific) equipped with the Fluofit software for lifetime evaluation.

### Synthesis of the Investigated Triazine I

**Scheme 1:**
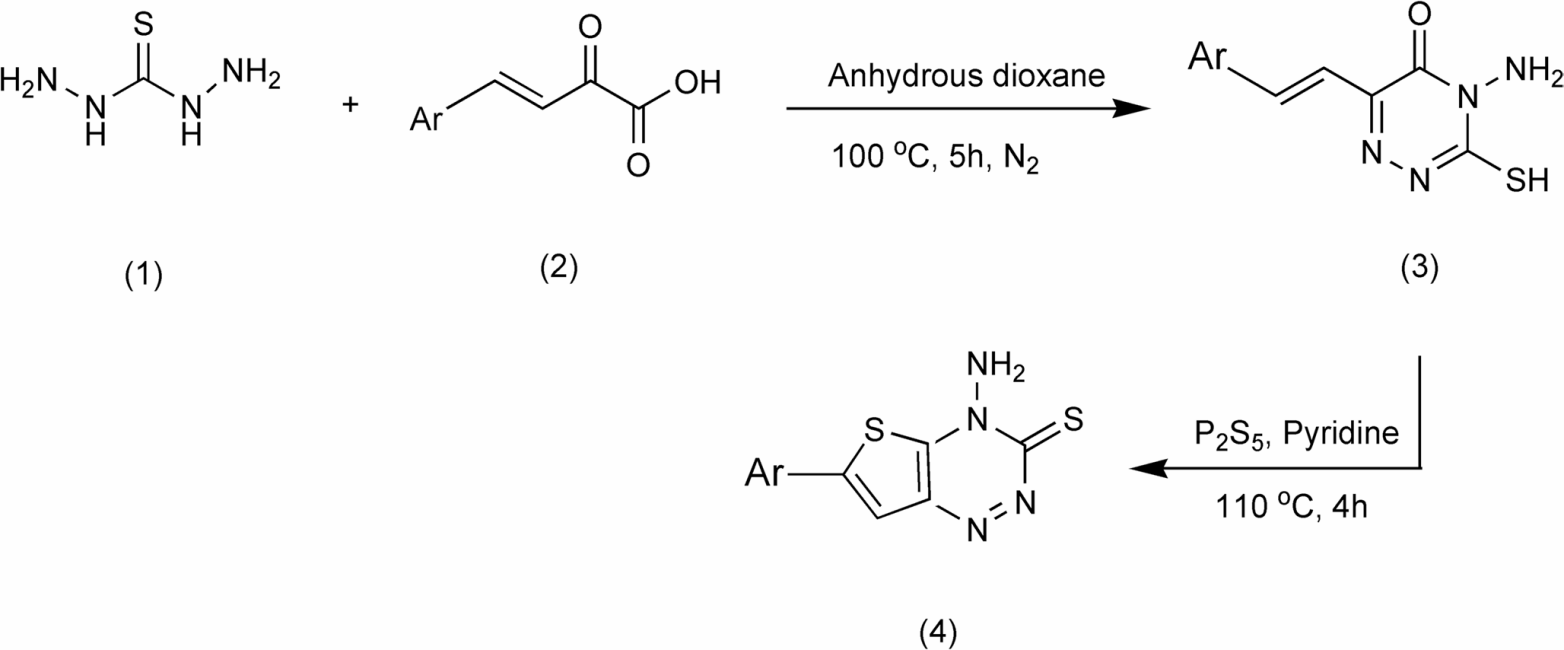
Preparation of (Triazine I; compound 4) (Ar = MeOC_6_H_5_)

Triazine I dye was synthesized by using the previously described general condensation process via two steps that involve the sequential condensation of thiocarbohydrazide with arylidenepyruvic acid, followed by cyclization using phosphorus pentasulfide [21]. A detailed discussion of the synthetic methodology of Triazine I is given in the supporting information, and the synthetic route is outlined in Scheme 1. The structure of the compound was confirmed by FT-IR spectra (Fig. S1), EI-mass (Fig. S2), ^1^H-NMR (Fig. S3), and ^13^C-NMR spectra (Fig. S4). The physical and spectral criteria of Triazine I are: Yield 78%, reddish orange crystals, m.p.: 312–315 ^o^C; (EI) m/z: calcd for [C_12_H_12_N_4_OS_2_] 292.37; found, 292.38; IR(KBr): ν = 1631 (CS), 1598 (CN), 2959 (CH), 3241 (NH_2_) cm^−1^; ^1^HNMR (DMSO-d_6_): δ (ppm) 3.53 (s, 3 H, OCH₃), 3.81 (s, 3 H, OCH₃), 4.25 (br s, 2 H, NH₂), 6.49 (s, 1H, Ar–H), 6.94 (d, J = 7.8 Hz, 1H, Ar–H), 7.05 (d, J = 8.4 Hz, 1H, Ar–H), 7.58 (d, J = 8.7 Hz, 1H, Ar–H), 7.75 (s, 1H, Ar–H), 7.80 (d, J = 8.1 Hz, 1H, Ar–H); 13CNMR: δ (ppm) = 160.2 (C = S), 158.4 (C-OCH₃), 128.99 (Ar-C), 115.44 (Ar-C), 100.0 (Ar-C), 55.3 (OCH₃), 40.41 (CH₂); C_12_H_12_N_4_OS_2_ (292.38); Calcd: C, 49.30; H, 4.14, N, 19.16. Found: C, 49.14; H, 4.11; N, 19.08.

Quinine sulfate as standard reference material in an optically diluted solution (Φ_F_ = 0.54) in 0.5 mol dm^−3^ H_2_SO_4_ was used to estimate quantum yields of fluorescence (Φ_F_) [22]. Reabsorption was minimized by using low concentrations of the sample ($\:\le\:\:$0.1 absorbance unit). The quantum yields of fluorescence were calculated according to Eq. (1)

1 $${\left({\Phi }_{F}\right)}_{s}={\left({\Phi }_{f}\right)}_{r} \times \frac{{I}_{S}}{{I}_{r}}\times \frac{{A}_{r}}{{A}_{s}} \times \frac{{n}_{s}^{2}}{{n}_{r}^{2}}$$

Where (Φ_F_) _s_ and (Φ_F_) _r_ are the quantum yields of fluorescence of both sample and reference, respectively, I is the integrated emission intensity, A is the absorbance at the wavelength of excitation, and n is the solvent’s refractive index.

### Sample Preparation

The samples were prepared in 5 mL measuring flasks containing 0.3 mL of 8.35 × 10^−5^ mol/L of Triazine I for UV-vis and fluorescence spectra measurements. The solvent effect was investigated using a variety of solvents, including cyclohexane, THF, dioxane, DMF, DMSO, propanol, ethanol, methanol, and ethylene glycol. To measure the fluorescence of dye samples, the excitation wavelength was modified based on the maximum wavelength of absorption.

### Synthesis of Metallic Nanoparticles

#### Silver NPs (Ag-NPs) Synthesis

Ag-NPs were synthesized via citrate reduction of AgNO_3_ [23]. An aqueous solution of AgNO_3_ (1 mM, 125 ml) was heated to a boiling point (100 °C) with continuous stirring. Once the solution reached a boiling point, 5 ml of 1% trisodium citrate solution (as an agent for nucleation and reduction) was rapidly added. The solution’s color immediately changed to pale yellow, indicating the formation of silver nanoparticles. Upon observing the color change, the solution was removed from the heating source and left to stir at room temperature until it cooled to ambient temperature.

#### Synthesis of Gold NPs (Au-NPs)

Au-NPs were prepared by utilizing the citrate reduction of HAuCl_4_.3H_2_O [24, 25]. An aqueous solution of HAuCl_4_.3H_2_O (1 mM, 100 mL) was brought to reflux while stirring, and then 10 mL of a 1% trisodium citrate solution (as nucleating and reducing agent) was added quickly, which resulted in a change in solution color from Pale yellow to deep red. After the color change, the solution was refluxed for an additional 15min and allowed to cool to room temperature. The literature provides a detailed explanation of the process underlying the sequential reduction of [AuCl_4_]^−^ ions to produce metallic AuNPs [26, 27].

### Computational Methodology

Through the utilization of the CAM-B3LYP [28] level and a 6- 311 G++ (d, p) [29] basis set, density functional theory (DFT) methods [30–38] were used to investigate the photoelectronic characteristics and molecular modeling of Triazine I in a variety of solvents. The Gaussian 16 software was applied to illustrate all computational calculations [39]. By using DFT/CAM-B3LYP/6-31G++ (d, p) level, the Triazine I molecular structure in various solvents is promoted, and their molecular electronic characteristics, including the energy gap (E_g_) and HOMO, LUMO levels, are acquired. The electrostatic potential and the spectra of electronic absorption computed for Triazine I in all investigated solvents are calculated by applying the CAM-B3LYP/6-31G++ (d, p) level of theory [40]. The calculated electronic absorption spectra are calculated using time-dependent density functional theory (TD-DFT) [41, 42]. The partial density of states for all studied molecular structures is calculated using Multiwfn software [43].

## Results and Discussion

### Computational Investigations

#### Optimized Molecular Structure in Different Solvents

**Fig. 2:**
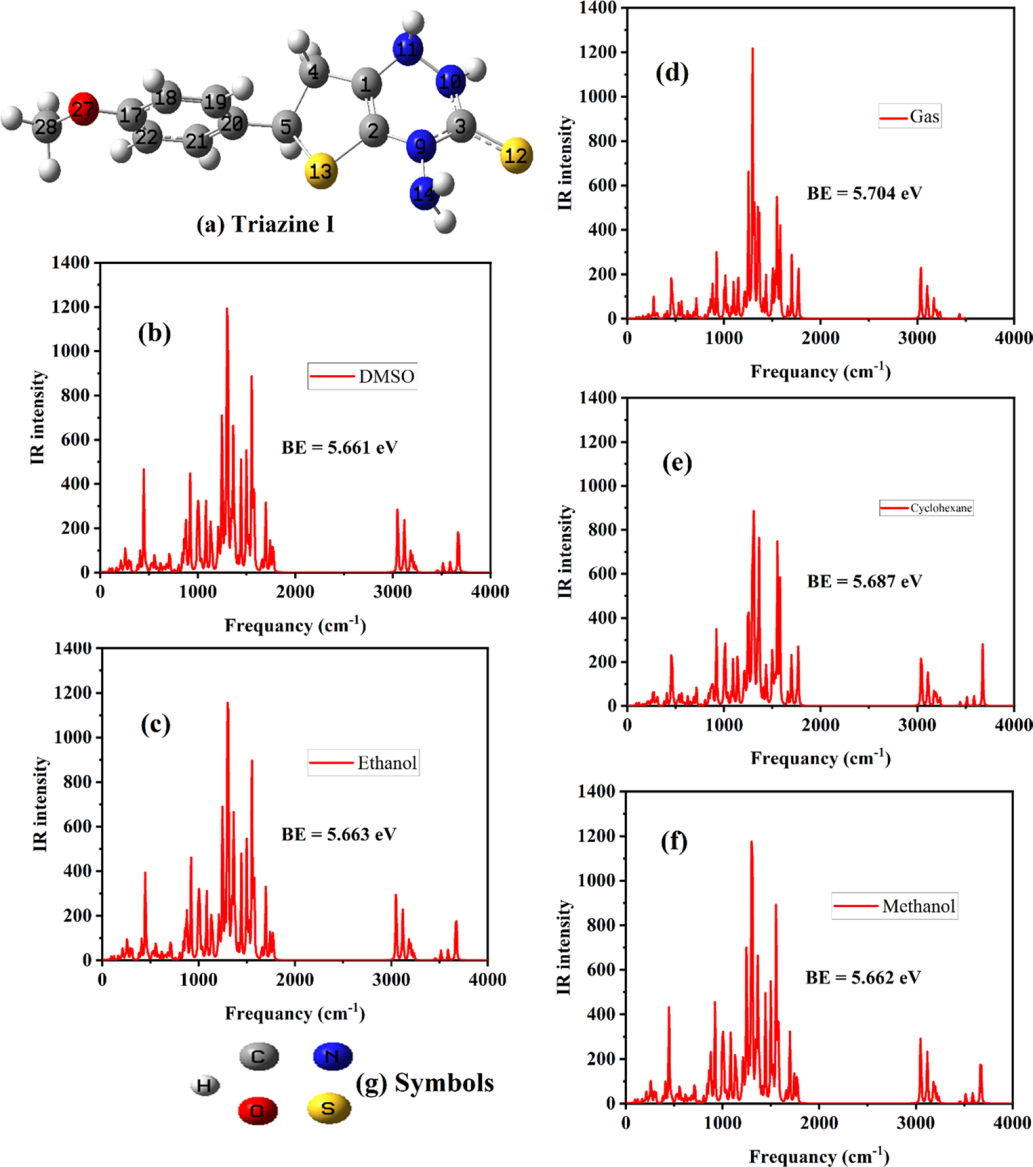
Optimized structure of Triazine I (**a**). IR and BE for Triazine I in different solvents in (**b**-**f**). The symbol color of the atoms used in Triazine I is (**g**)

The structural parameters of Triazine I were investigated across different solvents, as depicted in Fig. 2 and detailed in Table 1. Figure 2 and Fig. S5 present the optimized structure of Triazine I in both the gaseous phase and various solvents, including methanol, ethanol, DMSO, and cyclohexane. These figures visually depict the molecular arrangement, offering insights into its structural adaptations under different conditions (referenced in Fig. 2). Meanwhile, Table 1 presents important quantum parameters such as bond lengths, bond angles, and dihedral angles for Triazine I in various solvents, highlighting the influence of solvent environment on its structural properties (citation: Table 3). Analysis of the data reveals solvent-dependent variations in Triazine I’s molecular geometry. For instance, bond lengths, such as C1-N11 and C2-N9, show minimal differences across solvents, indicating structural stability Table 1 . However, variations in bond angles, such as C1-N11-N10 and C4-C1-C4, suggest solvent-induced conformational changes Table 1 . In methanol and ethanol, for instance, the C2-C1-C4 angle deviates notably compared to other solvents, indicating a solvent-specific effect on molecular geometry. These solvent-induced changes can be attributed to solvent polarity and interactions with Triazine I. Polar solvents like methanol and ethanol may form stronger hydrogen bonds with Triazine I, influencing its molecular conformation compared to nonpolar solvents like cyclohexane. This is evident in the dihedral angles involving nitrogen atoms, which exhibit larger deviations in polar solvents compared to nonpolar ones Table 1 . Despite solvent effects, Triazine I maintains overall stability across solvents, which is crucial for its potential applications. Understanding these solvent-dependent variations is essential for tailoring Triazine I’s properties to specific applications. For example, in drug delivery systems, the choice of solvent can affect the encapsulation efficiency and release kinetics of Triazine I-based carriers. In summary, the structural analysis of Triazine I in different solvents reveals solvent-dependent variations in its molecular geometry, influenced by solvent polarity and interactions. These findings underscore the importance of considering solvent effects when designing and optimizing Triazine I-based materials for various applications.

**Table 1:** Some significant quantum parameters such as bond length (Å), dihedral, and bond angles (degrees) for Triazine I in different solvents

Designations	Gas	Methanol	Ethanol	DMSO	Cyclohexane
C1-N11	1.429	1.428	1.428	1.428	1.429
C2-N9	1.400	1.403	1.402	1.403	1.401
C3-S12	1.666	1.684	1.683	1.684	1.673
C5-C20	1.503	1.503	1.503	1.503	1.503
C17-O27	1.355	1.355	1.355	1.355	1.355
C1-N11-N10	108.988	108.853	108.854	108.851	108.922
C2-C1-C4	118.654	115.62	115.616	115.623	115.487
C21-C20-C19	117.875	117.928	117.927	117.928	117.895
C4-C1-N11-N10	153.366	154.414	154.389	154.439	153.6
C19-C20-C5-C4	62.366	57.99	58.093	57.886	60.767
S13-C2-N9-N14	6.577	9.349	9.293	9.406	7.675

#### Stability Investigations in Different Solvents

The stability of Triazine I in gas, methanol, ethanol, DMSO, and cyclohexane solvents was studied by determining the binding energy (BE) and utilizing frequency calculations. BE was determined using the equation [44]: BE = (N_C_ E_C_ + N_H_ E_H_ + N_N_ E_N_ + N_O_ E_O_ + E_S_ N_S_ - E_t_)/N_t_, where N_C_, N_H_, N_N_, N_O_, N_S_, and N_t_ represent the numbers of C, H, N, O, S, and the total number of atoms, respectively. E_C_, E_H_, E_N_, E_O_, E_S_, and E_t_ denote the corresponding total energies of C, H, N, O, S, and the final compound, respectively. The calculated binding energies, ranging from 5.661 to 5.704 eV, consistently indicate the stability of Triazine I in various solvents. These values are depicted in Fig. 2 (b-f), illustrating Triazine I’s stability across different solvents. Notably, modifications made to Triazine I in various solvents influenced the binding energy. Specifically, Triazine I in DMSO exhibited lower binding energies compared to Triazine I in gas and the other solvents studied, suggesting enhanced reactivity in DMSO due to its high polarity. To further evaluate the dynamical stability of Triazine I in various solvents, we evaluated the infrared (IR) spectra obtained from the calculations of frequency. The actual vibrational frequencies presented in Fig. 2 (b-f) verify that the potential energy surface does not have saddle points, indicating the triazine I’s dynamic stability in various solvents. The selection of these solvents was based on their usage in experimental sections, facilitating comparison between computational and experimental results. This comprehensive analysis provides insights into the stability and characteristics of Triazine I across different solvent environments, essential for its potential applications.

#### Electronic Investigations

**Fig. 3:**
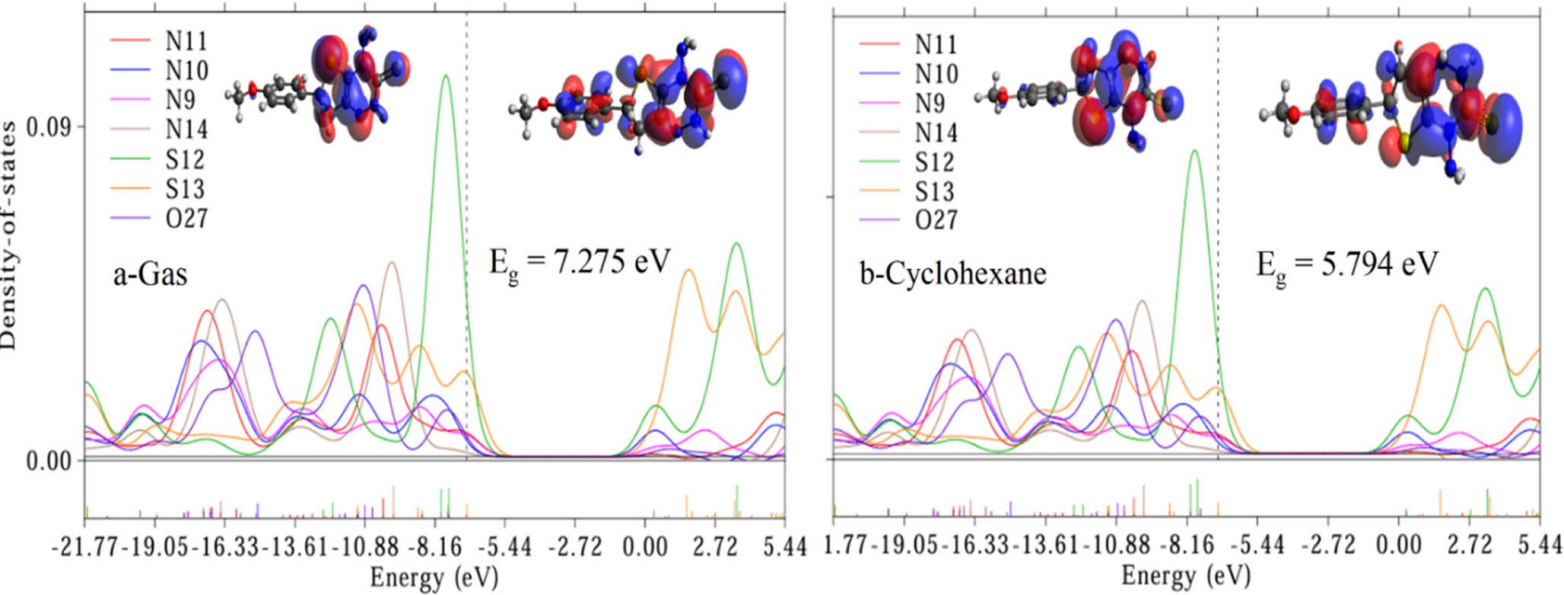
Partial density of states (PDOS) for hetero atoms of Triazine I in gas and cyclohexane respectively, and the corresponding HOMO/LUMO graps. The vertical dotted line is the Fermi level. The HOMO lies on the left of Fermi level and the LUMO lies on the right of the Fermi level

**Fig. 4:**
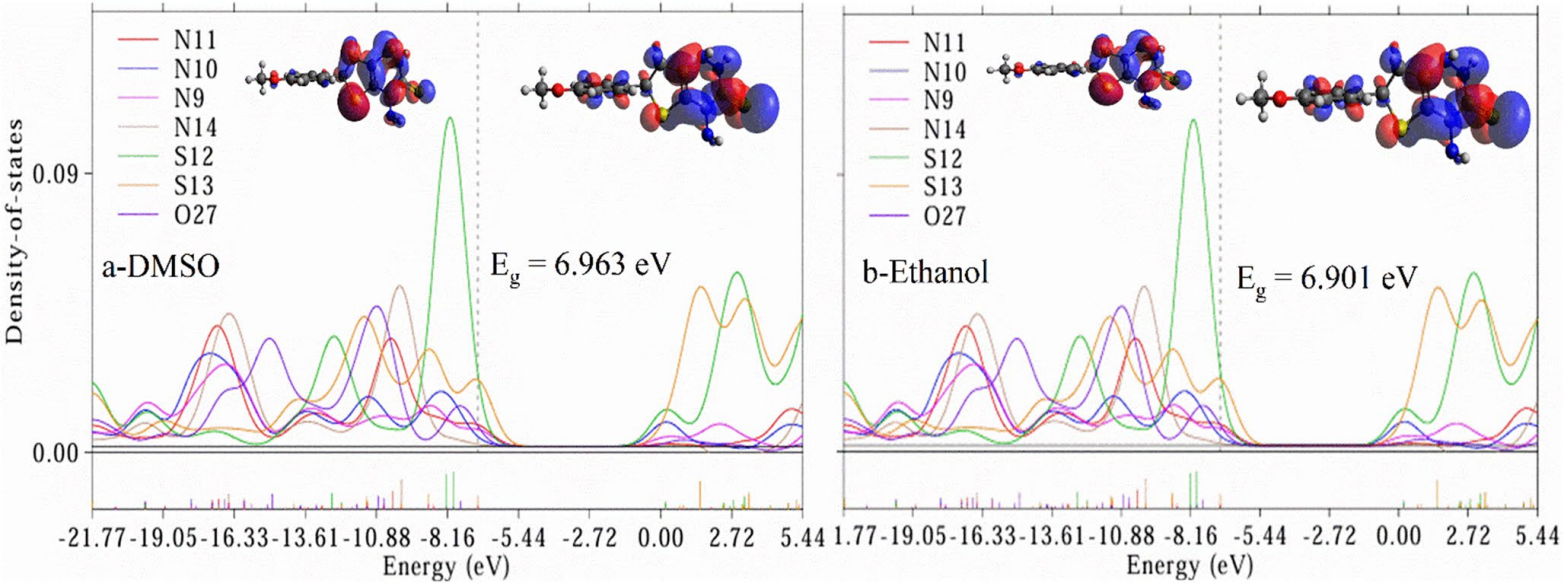
Partial density of states (PDOS) for hetero atoms of Triazine I in DMSO and ethanol respectively, and the corresponding HOMO/LUMO graps. The vertical dotted line is the Fermi level. The HOMO lies on the left of Fermi level and the LUMO lies on the right of the Fermi level

**Fig. 5:**
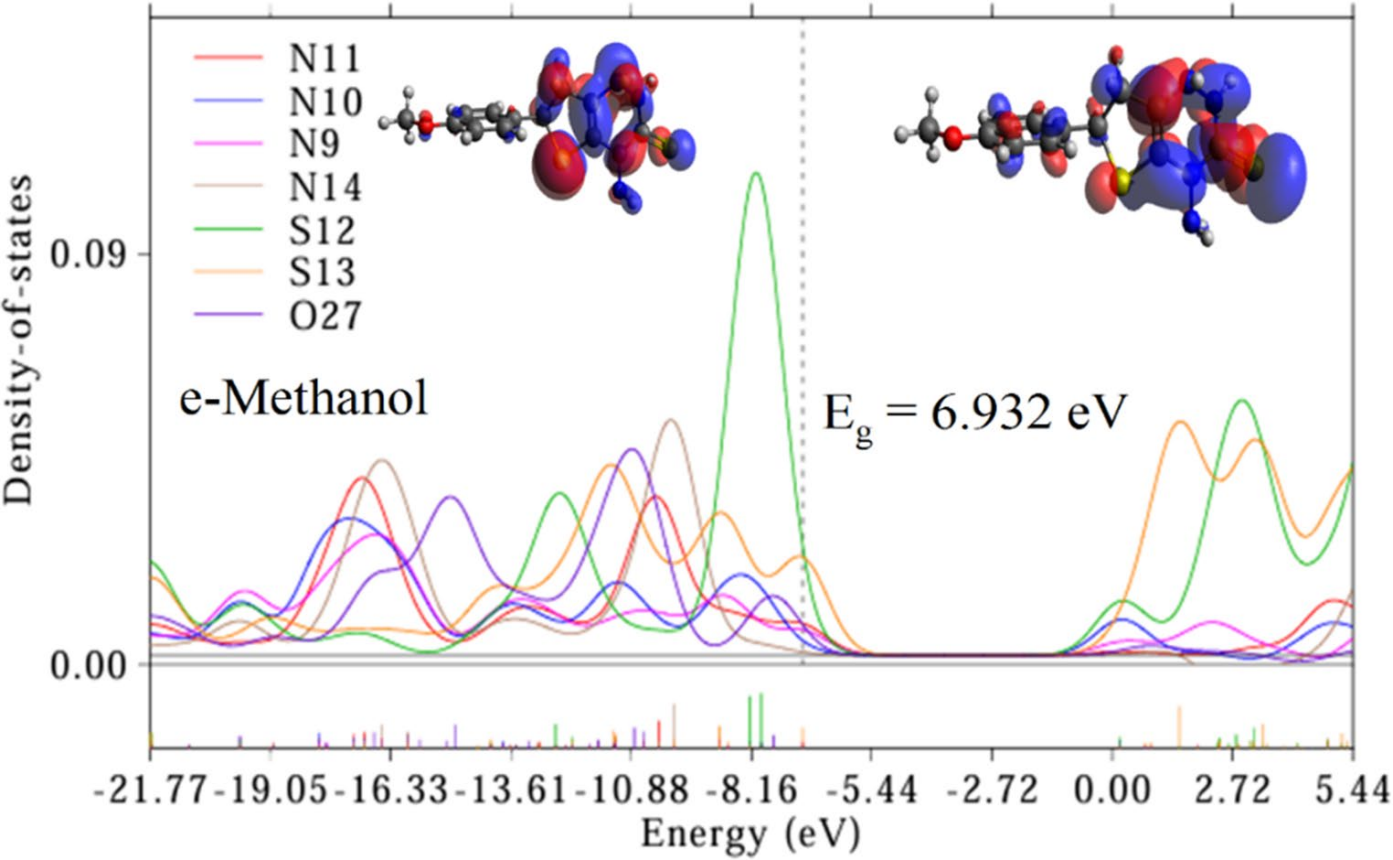
Partial density of states (PDOS) for hetero atoms of Triazine I in methanol and the corresponding HOMO/LUMO graps. The vertical dotted line is the Fermi level. The HOMO lies on the left of Fermi level and the LUMO lies on the right of the Fermi level

To investigate the electronic properties of Triazine I, we employed partial density of states (PDOS) analysis and examined the highest occupied/lowest unoccupied molecular orbitals (HOMO/LUMO). The PDOS and (HOMO/LUMO) spectra, depicted in Figs. 3, 4 and 5 were obtained through analysis of the Gaussian output file using Multiwfn software, which calculates the percent contribution of each heteroatom to the molecular orbitals. The overlapping PDOS spectra suggest strong orbital interactions and electron delocalization within the molecule. Notably, higher PDOS peaks were consistently observed for S12 across all studied solvents compared to other hetero atoms in Triazine I, indicating S12 as the primary electron donor. These findings mechanistically justify the fluorescence quenching by Ag-NPs, which likely interact with S-based donor regions to facilitate nonradiative decay. Conversely, the Au-NP-induced fluorescence enhancement may result from plasmonic field effects near the LUMO-localized phenyl region. The HOMO primarily localizes electrons on the triazine and tetrahydrothiene rings, while the LUMO, representing the lowest energy level available for electron acceptance, localizes electrons on the triazine, tetrahydrothiene, and phenyl rings.

Consequently, the triazine and tetrahydrothiene rings act as electron-donating groups, while the phenyl ring serves as an electron-accepting group. This orbital distribution supports the experimentally observed ICT emission: upon excitation, electron density shifts from the electron-rich donor region (triazine/S) to the electron-acceptor region (phenyl ring). The result is a red shift in emission in polar solvents, consistent with stabilization of the charge-separated excited state, as seen in Fig. 7b. The energy gap (E_g_) of Triazine I in various solvents was calculated using the formula E_g_ = E LUMO – E HOMO. The corresponding E_g_ values are presented in Figs. 3, 4 and 5. Here, E LUMO and E HOMO refer to the energies of the highest occupied and lowest occupied molecular orbitals, respectively. the HOMO and LUMO energy respectively. A significant E_g_ value was observed in the gas solvent (7.275 eV), whereas a lower value was observed in the cyclohexane solvent (5.794 eV) due to the non-polarity of cyclohexane compared to gas and other polar solvents. This observation underscores the influence of solvent polarity on the energy gap of Triazine I, affecting its electronic properties and potential applications. In summary, the investigation into Triazine I’s electronic properties via PDOS analysis and HOMO/LUMO examination revealed significant electron delocalization, with S12 identified as the primary electron donor. The energy gap calculations underscored the solvent-dependent variations, emphasizing the importance of solvent polarity in modulating Triazine I’s electronic behavior.

#### MEP Investigations

**Fig. 6:**
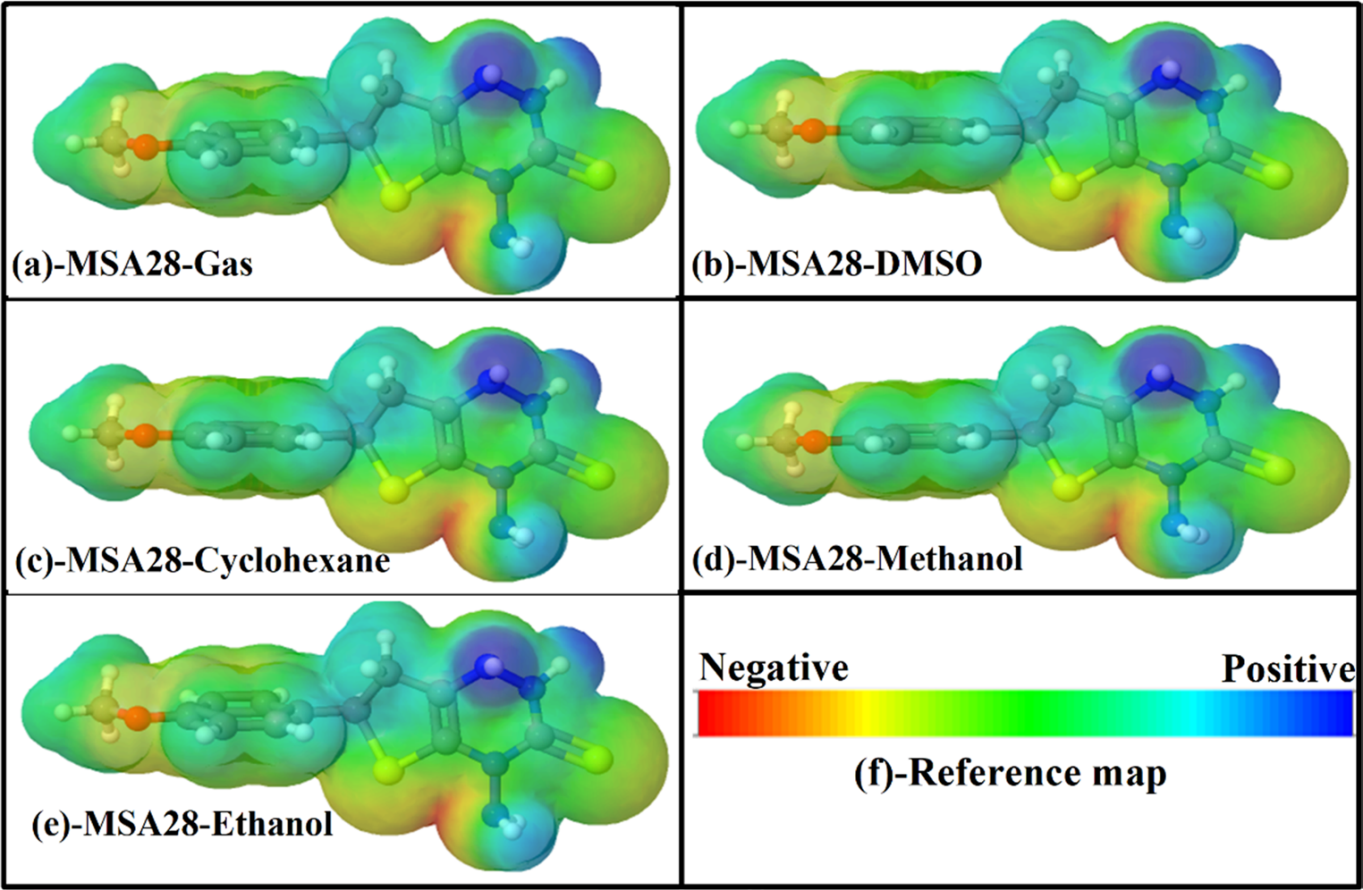
Molecular electrostatic potential (MEP) surfaces for Triazine I in various solvents (**a**-**e**). The isodensity surfaces are displayed at a value of 0.004 e/Bohr³, and the electrostatic potential values are represented in atomic units (a.u.). (**f**) Reference map indicating the color scale, where red denotes regions of most negative potential and blue indicates regions of most positive potential

A molecular electrostatic potential (MEP) analysis was conducted to discern the electrophilic and nucleophilic sites within Triazine I across various solvent environments [45]. Figure 6 (a-e) depicts the MEPs, employing a color scheme that delineates differing charge densities: red denotes regions with an electron-rich, partially negative charge; blue indicates regions with an electron-deficient, partially positive charge; light blue highlights slightly electron-deficient regions; yellow signifies slightly electron-rich areas, and green represents neutral (zero potential) regions (please refer to Fig. 6 (a-e)). Consistently across all studied solvents, the S12 and S13 atoms are enveloped by a negative region (depicted in yellow), manifesting as electron-rich and thus susceptible to electrophilic attacks within the Triazine I surface. These MEP predictions strongly support the experimental observation that Ag and Au nanoparticles, particularly those forming thiol-type interactions, bind preferentially at these S sites. This explains why fluorescence quenching is most pronounced in solvents like ethylene glycol, where sulfur-nanoparticle interactions are enhanced by viscosity and polarity.

Conversely, the hydrogen atoms bonded to nitrogen atoms (N-H) exhibit a positive region (depicted in blue) in the MEP, indicative of electron deficiency and predisposition to nucleophilic attack. In summary, the MEP analysis of Triazine I in various solvents reveals electron-rich regions surrounding S12 and S13 atoms, rendering them susceptible to electrophilic attacks, while the N-H bonded hydrogen atoms exhibit electron-deficient regions, making them prone to nucleophilic attack.

#### Optical Investigations

Figure S6 illustrates the computational electronic absorption spectra for Triazine I in various solvents, revealing distinct peaks indicative of electronic transitions from the ground state to excited states. The solvent environment influences these transitions, as evidenced by differences in peak positions and intensities. Upon examination of Table 2, notable variations in the theoretical maximum wavelength (λ_Th_.) and experimental maximum wavelength (λ_exp_.) across solvents are observed. For instance, in methanol, the theoretical maximum wavelength for the highest excited state (ES 7) is calculated to be 233.33 nm, while the experimental maximum wavelength is observed at 354.4 nm. This discrepancy between theoretical and experimental values suggests solvent-induced shifts in electronic transitions. The comparison between theoretical and experimental optical properties reveals both strong correlations and expected discrepancies. Qualitatively, our computational results align with experimental observations regarding the impact of solvent polarity. For example, both experimental UV-vis absorption and fluorescence spectra showed a significant redshift in emission spectra with increasing solvent polarity, suggesting a higher dipole moment in the excited state compared to the ground state. The computational electronic absorption spectra also demonstrate that solvents with higher polarity induce redshifts in absorption peaks, increase oscillator strengths, and stabilize electronic transitions, which is in general agreement with the experimentally observed solvent-dependent behaviour. This consistency underscores the ability of our computational model to capture the general trends of solvent-solute interactions and their influence on the electronic transitions of Triazine I. However, as noted in Table 2, quantitative differences are observed between the theoretical and experimental absorption maxima (e.g., 233.33 nm theoretical vs. 354.4 nm experimental in methanol). These discrepancies are not uncommon in integrated experimental-theoretical studies and can be attributed to several factors. Firstly, the computational models, such as implicit solvent models used here, may not fully account for all specific solvent-solute interactions, including explicit hydrogen bonding or dynamic solvent rearrangement, which play a significant role in solution-phase spectroscopy. Secondly, the experimentally observed absorption maximum might represent a complex interplay of multiple electronic transitions or a different dominant transition than the one predicted as the highest excited state in the theoretical calculations. Lastly, experimental measurements are subject to environmental factors and instrumental conditions that are idealized in computational models. Despite these quantitative differences, the theoretical calculations provide invaluable atomistic and electronic insights that are difficult to obtain experimentally, such as specific orbital contributions and transition likelihoods, thus complementing and deepening our understanding of Triazine I’s photophysical behaviour.

Furthermore, the oscillator strength (ƒ) and transition coefficient (TC) provide insights into the likelihood and strength of electronic transitions, respectively. In cyclohexane, for instance, the oscillator strength for the transition from the ground state to the first excited state (ES 1) is notably higher (0.082) compared to other solvents. This indicates a more favorable transition in cyclohexane due to its lower polarity compared to polar solvents like methanol and ethanol. Additionally, comparing the transition energies (TE) across solvents highlights the influence of solvent polarity on electronic transitions. For instance, in DMSO, the transition energy for the highest excited state (ES 7) is calculated to be 4.1274 eV, whereas in ethanol, it increases to 5.3121 eV. This shift towards higher energies in polar solvents indicates a stabilization of the excited state due to stronger solvent interactions. In summary, the observed trends in both Fig. S6 and Table 2 underscore the significant influence of solvent polarity on the electronic absorption properties of Triazine I. Solvents with higher polarity induce redshifts in absorption peaks, increase oscillator strengths, and stabilize electronic transitions. These findings are crucial for understanding the molecular behavior of Triazine I in solution environments and have implications for various applications in spectroscopy and molecular design.

**Table 2:** The calculated excited state (ES), theoretical maximum wavelength (λ_Th_.), experimental maximum wavelength (λ_exp_.), transition energy (TE), electronic transition (ET), oscillator strength (ƒ), and transition coefficient (TC)for Triazine I in various solvents

Solvents	ES	λ_Th_.(nm)	λ_exp_(nm)	TE (eV)	ET	f	TC
Gas	7	229.58	-	5.4004	H→L + 4	0.321	0.300
	2	286.62			H→L	0.056	0.650
Methanol	7	233.33	354.4	5.1561	H→L	0.416	0.170
	1	300.01		4.1327	H→L	0.067	0.663
Ethanol	7	233.4	357	5.3121	H→L	0.423	0.203
	1	299.96		4.1334	H→L	0.068	0.413
DMSO	7	233.44	346	4.1274	H→L	0.413	0.664
	1	300.39		5.3111	H→L	0.070	0.158
Cyclohexane	7	234.48	358.5	5.2875	H→L	0.396	0.157
	1	296.65		4.1794	H→L	0.082	0.265

### Solvent Effect on Triazine I Optical Spectra

**Fig. 7:**
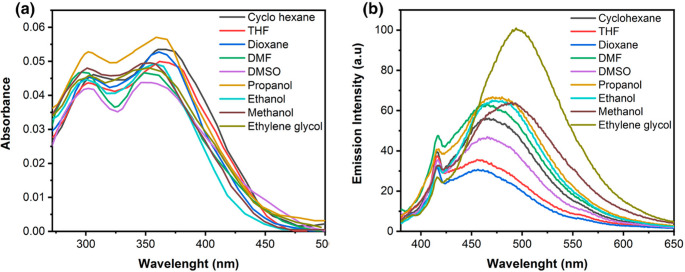
(a) and (b) Absorption and emission spectra of (5 × 10^−6^) M of Triazine I in different solvents, respectively, (λ_ex._ = 370 nm)

The UV-vis absorption and fluorescence spectra of 5 × 10^−6^ mol L^−1^ of Triazine I recorded in solvents of different polarities are shown in Fig. 7 (a and b). Some important photophysical parameters such as absorption (λ_abs_.) and emission (λ_em_.) maxima, the Stokes shifts in wave numbers $\left(\Delta\overline v\right)$ in cm^−1^, extinction coefficients (ε) in M^−1^ cm^−1^, fluorescence quantum yield (Φ_f_), oscillator strength (ƒ), the transition dipole moment (µ_12_) in Debye, solvent polarity parameters ($\:{E}_{N}^{T}$), and orientation polarizability (Δƒ) are shown in Table 3 . As noticed in Fig. 7 (a), Triazine I shows two broad bands of absorption on going from DMF to ethylene glycol, and the solvent polarity has minimal effect on the absorption maxima, indicating the weak polar character of Triazine I in the ground state. But in Fig. 7 (b), on excitation at 370 nm, its emission spectra exhibit two bands arising from the locally excited state and the state of intramolecular charge transfer (ICT), respectively [46].

When the solvent polarity is changed from THF (polar aprotic solvent) to ethylene glycol (polar protic solvent), there is a significant redshift in the emission spectra from 455.5 to 496 nm. However, the absorption spectrum does not show a similar shift. In such cases, the relaxed excited state S_1_ will be energetically stabilized relative to the ground state S_0_, and a significant redshift of the fluorescence will be observed. The pronounced shift in emission indicates that the dipole moment of the excited state is higher compared to that in the ground state [47]. The less pronounced shift in the absorption spectra observed in all the studied solvents implies that the ground-state energy distribution is not affected to a greater extent. Therefore, there is a noticeable variation in the Stokes shift’s magnitude, ranging from 5747 to 8087 cm^−1^, suggesting that the Triazine I excited state geometry may differ from its ground state geometry. It is found that the spectra of emission have a higher sensitivity to solvent polarity in comparison to absorption spectra, indicating that in the singlet excited state, photo-induced intramolecular charge transfer (ICT) takes place from the electron-donating substituent group to the electron acceptor group of the chromophore.

The fluorescence efficiency of the dye was significantly influenced by solvent polarity and viscosity. Among all tested solvents, the lowest fluorescence intensity was observed in 1,4-dioxane, a moderately polar aprotic solvent. In contrast, ethylene glycol, a highly polar protic solvent with strong hydrogen-bonding ability via its hydroxyl groups, resulted not only in the highest fluorescence intensity but also induced a noticeable red shift in the emission wavelength (λ_f_ = 496 nm). This shift is attributed to strong hydrogen bonding interactions between the solvent’s diol groups and the dye’s electronegative nitrogen atoms, which stabilize the excited state and lower its energy.

Interestingly, although DMSO has a higher polarity index than DMF (0.444 vs. 0.404), the fluorescence intensity in DMSO was lower. This reduction can be attributed to DMSO’s higher viscosity (1.99 cP vs. 0.92 cP), which may hinder molecular motion and promote non-radiative decay, reducing fluorescence. In protic solvents such as propanol, ethanol, methanol, and ethylene glycol (with polarity indices of 0.617, 0.654, 0.762, and 0.792, respectively), a gradual increase in fluorescence intensity was observed with increasing polarity [48]. This trend supports the dominant role of solvent polarity in enhancing fluorescence, while viscosity appears to exert a secondary influence in some cases.

**Table 3:** Spectral and photophysical parameters of Triazine I in different solvents

Solvent	λ_max_(abs.) (nm)	λ_max_(em.) (nm)	Δ$\:\overline{v}\:$(cm^−1^)	ε M^−1^ cm^−1^	Φ_f_	$\:{E}_{T}^{N}$	(ε) Dielectric	*N*	Δƒ(D, *n*)	ƒ	µ_12_ Debye
Cyclohexane	358.5	467.5	6503.6	7911	0.015	0.006	2.02	1.4235	0.008	0.23	4.05
Dioxane	357.5	456.5	6066.2	7867	0.008	0.164	2.25	1.4224	0.024	0.22	3.92
THF	361	455.5	5746.9	7422	0.012	0.207	7.58	1.4072	0.210	0.228	3.99
DMF	348.5	465.5	7212.1	6933	0.022	0.404	36.71	1.4305	0.275	0.23	3.97
DMSO	346	465.5	7419.5	6489	0.019	0.444	46.68	1.479	0.263	0.213	3.83
Propanol	359	470.5	6601.2	8497	0.023	0.617	20.1	1.3856	0.274	0.249	4.17
Ethanol	357	480.5	7199.5	7289	0.022	0.654	24.55	1.3614	0.288	0.219	3.9
Methanol	354.5	490	7800.6	7333	0.017	0.762	32.6	1.326	0.308	0.237	4.06
Ethylene glycol	354	496	8087.3	7067	0.025	0.792	37	1.43	0.274	0.244	4.12

The simplified Lippert-Mataga’s Eqs. (2,3) [49, 50] method was utilized to estimate the dipole moment’s change (Δµ = µ_e_ – µ_g_) between Triazine I’s excited singlet state and ground state.

2 $$\Delta \overline{\nu } =\frac{{2({\mu }_{e}-{\mu }_{g})}^{2} }{hca} \Delta f+ const.$$

3 $$\Delta f = \frac{(\varepsilon -1)}{(2\varepsilon +1)}-\frac{\left({n}^{2}-1\right)}{{(2n}^{2}+1)}$$

Where $\Delta\overline v$ represents the difference between the maxima of absorption and emission in wave numbers (cm^−1^). The Planck constant is denoted by h; c is the light speed in vacuum; (a) is the cavity radius of Onsager. The solvent’s refractive index and dielectric constant are symbolized by n and ε, respectively; µ_e_ and µ_g_ refer to the excited and ground states’ dipole moments; and the solvent’s orientation polarizability is represented by Δƒ, which calculates the solvent molecule’s dipole moment and electron mobility. Suppan’s Equation is used to determine the Onsager cavity radius (a) based on the molecules’ molecular volume, Equation (4) [51].

4 $$a=\left(\frac{3M}{4\pi\;\delta N}\right)^\frac13$$

**Fig. 8:**
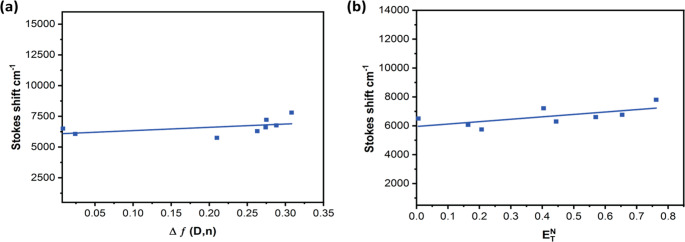
(**a**) Plot of Δƒ versus Stokes shift (cm^−1^) (**b**) Plot of $\:{E}_{T}^{N}$ versus Stokes shift (cm^−1^)

Where M and δ stand for the dye molecular weight and dye density, respectively, and N is Avogadro’s number. The magnitude of (a) for Triazine I was calculated to be 4.1 Å. The plot of the Stokes shift against the orientation polarization (Δƒ) can be seen in Fig. 8 (a). Based on this plot’s slope, the dipole moment change (Δµ) for Triazine I was estimated, and the calculated cavity radius (a) is 4.28 Debye. The atomic charges’ redistribution in the excited state brought about by transfer of charge from the groups that donate electrons to the groups that accept them was the source of this change in dipole moment. Reichardt’s solvatochromic shift method has been used to further study the difference between the two states, which yield (Δµ) [52–54]. The dimensionless microscopicsolvent polarity parameters ($\:{E}_{T}^{N})\:$is provided by Eqs. (5) and (6)

5 $$\:{E}_{T}^{N}\:=\:\frac{{E}_{T}\:\left(solvent\right)-30.7}{32.4}\:\:\:\:\:\:\:\:\:\:\:\:\:\:\:\:\:\:\:\:\:\:\:\:\:\:\:\:\:$$

6 $$\:{E}_{T}\left(\mathrm{s}\mathrm{o}\mathrm{l}\mathrm{v}\mathrm{e}\mathrm{n}\mathrm{t}\right)\:=\:\frac{28.591}{{}_{max}\left(nm\right)}$$

By establishing a correlation between the fluorophore’s Stokes shift and $\:{E}_{T}^{N}$, Δµ can be determined effectively using this method as shown in Fig. 8 (b), according to Eq. (7),

7 $${\nu }_{a}-{\nu }_{b}=11307.6 \left[{\left(\frac{\Delta \mu }{{\Delta \mu }_{B}}\right)}^{2}{\left(\frac{{a}_{B}}{a}\right)}^{3}\right]{E}_{T}^{N}+ const.$$

Where Δµ is the probe molecule’s dipole moment between the two states,

and Δµ_B_ is the dye’s dipole moment change; a is assumed to be 4.10 Å. Given that a_B_ and µ_B_ have known values of 6.2 Å and 9 Debye, respectively, Eq. (8) can be used to compute the change in dipole moment.

8 $$\Delta \mu ={\mu }_{e}-{\mu }_{g}=\sqrt{\frac{m\times 81}{{\left(\frac{6.2}{a}\right)}^{3}\times 11307.6}}$$

Where m is the linear plot’s slope of $\:{E}_{T}^{N}$ aganist Stokes shift as shown in Fig. 8 (b), and the Δµvalue is determined to be 1.86 D. Because only the dipole-dipole interactions are considered in Lippert’s plot and the polarizability of solute molecules is not taken into account, the value of Δµ calculated from the equation of Lippert-Mataga is larger than that gained using the solvent polarity parameters dimensionless microscopic $\:{\:E}_{T}^{N}$. The high Stokes shift values indicate that the geometry of the excited state differs greatly from the ground state geometry, leading to an enhancement in the dipole moment upon excitation.

In various solvents, the transition dipole moment (µ_12_) from the ground to the excited state of Triazine I was determined by Eq. (9) [55],

9 $${\mu }_{12}^{2}= \frac{f}{4.72 \times {10}^{-7}{E}_{max}}$$

Where E_max_ is the energy maximum absorption in cm^−1^ and *f* is the oscillator strength. The absorption area of the electronic spectrum is determined by the oscillator strength, which indicates the effective number of electrons undergoing transition from the ground to the excited state. The values of the experimental oscillator strength were estimated using Eq. (10) [56, 57].

10 $$f=4.32\times {10}^{-10}\int \varepsilon (\overline{\nu })d\overline{\nu }$$

Where ε is the molar decadic extinction coefficient’s numerical value in dm^3^ mol^−1^ cm^−1^ and 

$\:\overline v\:\:$is the wave number’s numerical value in cm^−1^. Table 3 lists the values of ƒ and µ_12_, which show that the S_0_ →S_1_ transition is firmly allowed.

As seen in Fig. S7 and Table 3, the polarity and hydrogen bonding ability of the various solvents have a significant impact on the dye fluorescence quantum yield (Φ_F_). Furthermore, Φ_F_ values increase and then decrease with increasing solvent polarity $\:{E}_{T}^{N}\:$that takes into account interactions like hydrogen bonding and solvent polarizability in addition to those of a particular kind. Significant mixing between the π-π* and n-π* close-lying states can be associated with effective intersystem crossing. Hydrogen bonds between solvent molecules explain the decrease in Φ_F_ in polar protic solvents (excluding ethylene glycol) caused by accelerated radiationless processes [58, 59].

### Metallic Nanoparticles’ Effect on Triazine I Optical Spectra

#### Characterization of Silver and Gold Nanoparticles

To describe the nature, size, shape, distribution, state of stability or aggregation, morphology, elemental composition, dispersity (monodisperse or polydisperse), and surface charge of nanoparticles, several analytical and spectroscopic techniques are employed [60].

#### A UV-vis Spectral Analysis

UV-vis spectroscopy is the primary tool that explains silver and gold nanoparticles’ formation at the initial synthesis stage. It is very characteristic depending upon the shape, size, and distribution of Ag and Au NPs due to surface plasmon resonance. The Ag-NPs exhibited a yellow color, which is characteristic of the chemical reduction and formation of Ag-NPs. While the prepared Au-NPs exhibited red color, similar to that reported [61].

**Fig. 9:**
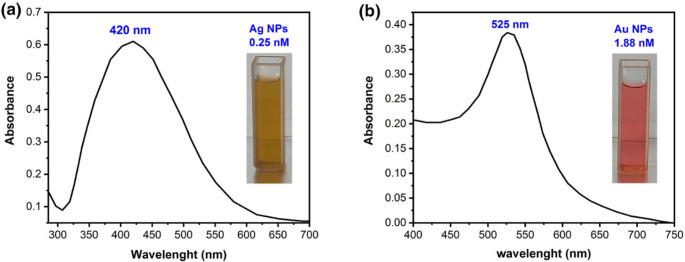
The UV-vis absorbance spectra of (**a**) silver nanoparticles and (**b**) gold nanoparticles

The absorption spectra of silver nanoparticle solution exhibit a significant SPR band around 420 nm with a diameter of 50 nm and a concentration of 0.25 nM, as shown in Fig. 9 (a). There is no absorption band for the pure silver nitrate solution (Ag). The absorbance spectra of gold nanoparticles revealed a distinctive surface plasmon band at ~ 525 nm with a diameter of 25 nm and a concentration of 1.88 nM, as shown in Fig. 9 (b), since the HAuCl_4_ does not have any absorbance at this wavelength. The wavelength of the plasmon absorption maximum in a given solvent can be used to indicate particle size.

#### B Transmission electron Spectroscopy (TEM)

**Fig. 10:**
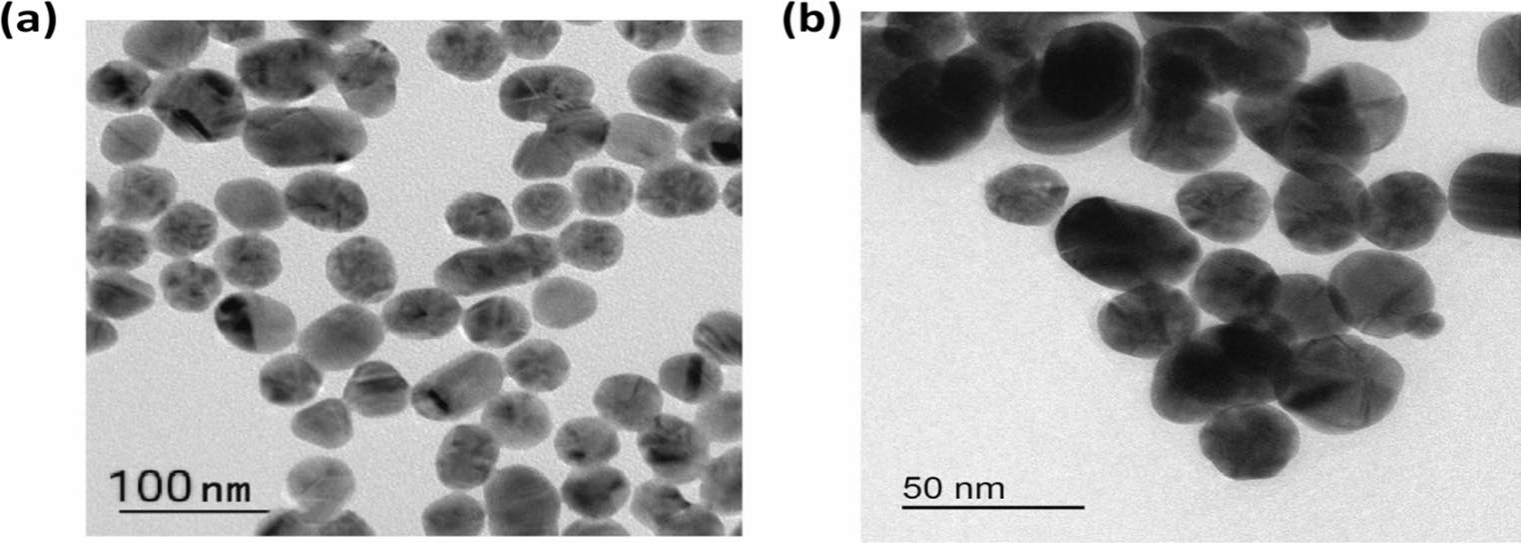
TEM image of (**a**) silver nanoparticles and (**b**) gold nanoparticles

Transmission electron microscopy (TEM) provides the most accurate and high-resolution imaging information about the size, shape, morphology, state of aggregation, and distribution of nanoparticles at nanometer resolution [62]. Figure 10(a) and (b) show the transmission electron micrographs of the freshly synthesized Ag and Au nanoparticles (50 nm and 25 nm in diameter, respectively), which were used in the experimental interaction described in this work. The particles exhibit a spherical shape.

#### C DynamiC Light Scattering (DLS)

**Fig. 11:**
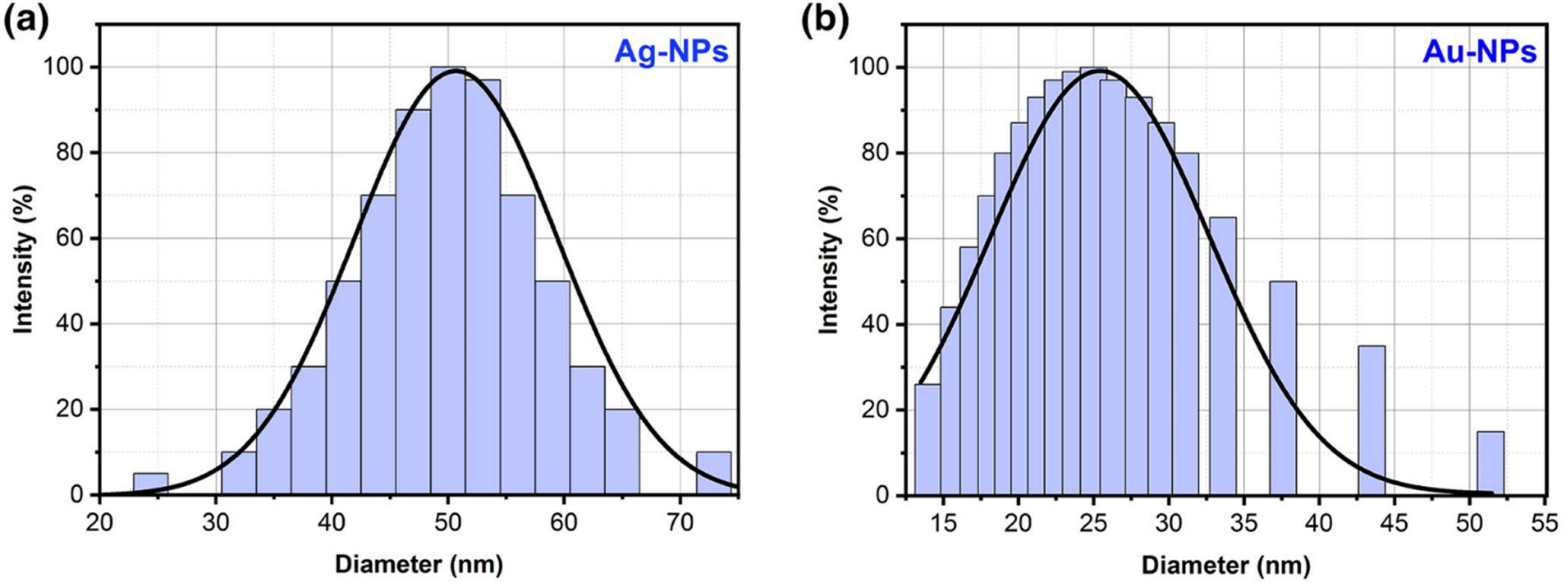
(**a**) Size distribution of Ag-NPs with average size 50 nm as analyzed using DLS, (**b**) Size distribution of Au-NPs with average size 25 nm as analyzed using DLS

Determination of particle size distribution can be done by dynamic light scattering. The graphs of the samples are shown in Fig. 11 (a) and (b). The particle size analysis (PSA) shows that the average particle sizes observed are around 50 nm and 25 nm for Ag-NPs and Au-NPs, respectively. The obtained nanoparticle size distribution of Ag-NPs and Au-NPs using DLS is also in good agreement with the TEM results.

#### D Zeta Potential (ZP)

**Fig. 12:**
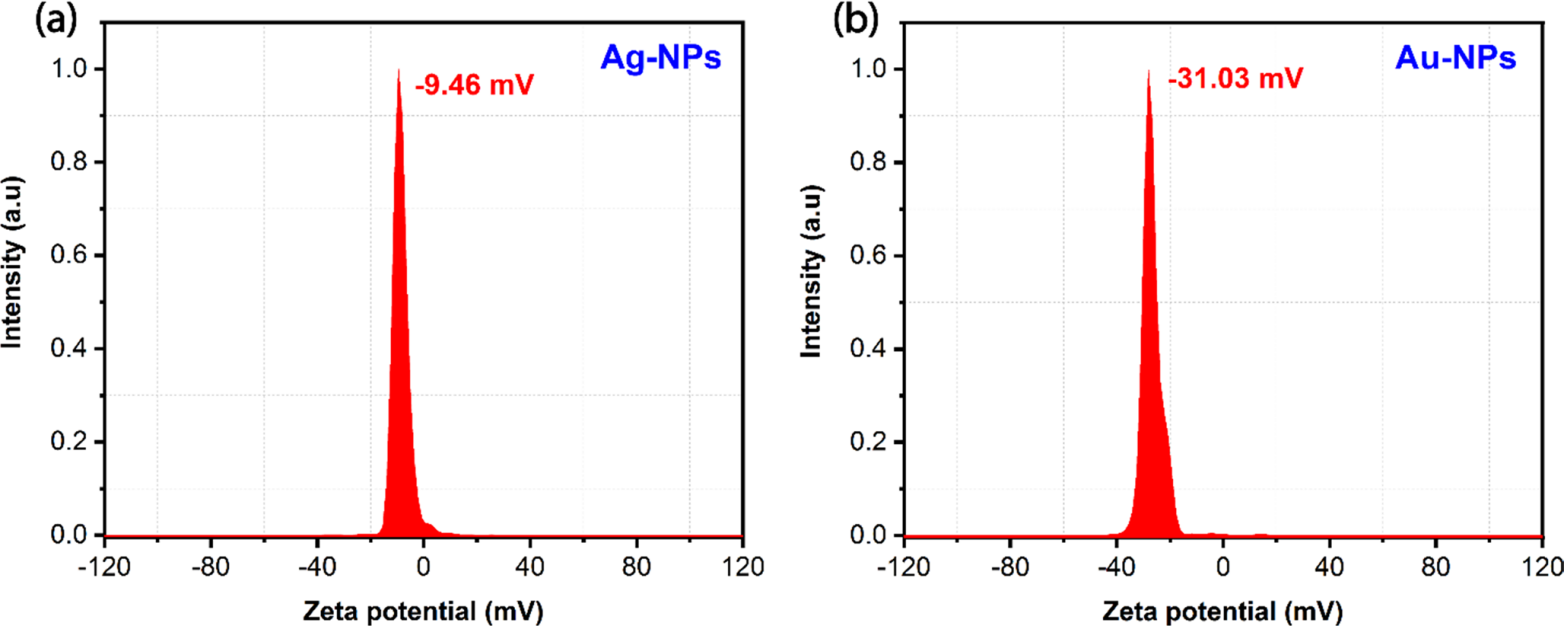
Zeta potential distribution of (**a**) Ag-NPs and (**b**) Au-NPs

Zeta potential analysis helps in understanding the nature of the forces that mediate interparticle interactions. The significance of zeta potential is that its value can be related to the stability of colloidal dispersions. As shown in Fig. 12 (a) and (b), the zeta potentials of Ag-NPs and Au-NPs were estimated to be −9.48 mV and − 31.03 mV, respectively, at pH 5.60. The negative values of Zeta potential for the samples indicate the minimum possibility for coagulation, which leads to enhanced colloidal stability over an extended period.

#### E Powder X-ray Diffraction

**Fig. 13:**
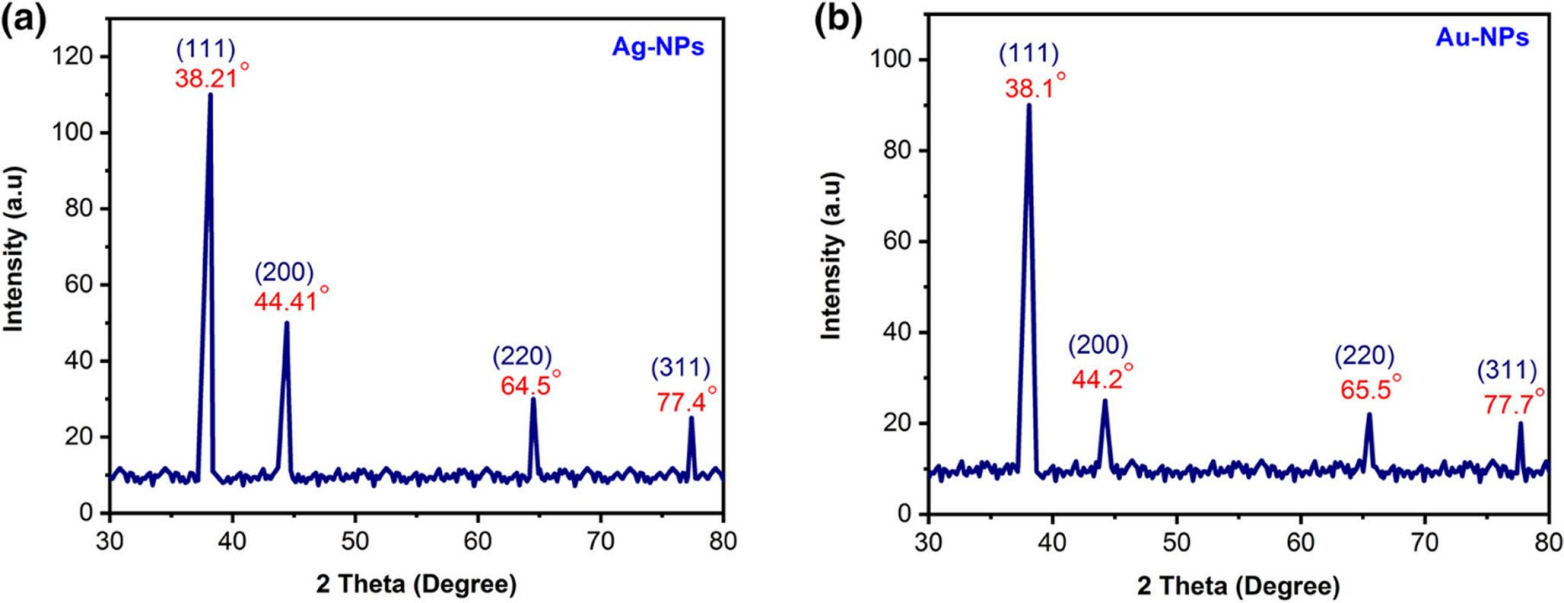
Powder X-ray diffraction plot of (**a**) Ag-NPs and (**b**) Au-NPs

The powder diffraction (XRD) patterns of the Ag-NPs and Au-NPs are shown in Fig. 13 (a) and (b) to identify their crystal structure. The XRD pattern of synthesized Ag/Au NPs was analyzed and compared to the standard powder diffraction card designed by the JCPDS. The characteristic crystalline nature of nanomaterials has been determined using the Debye–Scherrer formula.

$$D = K / \beta cos \theta$$

where D is the coherent scattering length (crystalline size in nm), K is the Scherrer’s constant (0.98), λ is the wavelength of the X-ray source, β is the angular FWHM of the XRD diffraction peak, and θ is the Bragg angle. Using Origin Pro 2023 software, the FWHM was computed from the Gaussian function.

According to the FCC structure of Ag-NPs, intense diffraction peaks attributable to Ag-NPs are observed at 2θ values of 38.21°, 44.41°, 64.5°, and 77.4°, which correspond to the (111), (200), (220), and (311) Bragg reflection planes, Fig. 13(a). The 2θ angles are measured in degrees (°), and the observed peaks are in good agreement with the standard data of face-centered cubic silver (JCPDS card No. 04-0783). The reflections were sharp and intense, demonstrating the highly crystalline character of the produced Ag-NPs. Similarly, the XRD patterns of Au-NPs show characteristic peaks at 2θ values of 38.1°, 44.2°, 65.5°, and 77.7° corresponding to the (111), (200), (220), and (311) Bragg’s reflection planes as illustrated in Fig. 13 (b). The 2θ angles are also measured in degrees (°), and the peaks match well with the standard data of face-centered cubic gold (JCPDS card No. 04-0784). Reflections were sharp and intense, indicating the crystalline nature of the produced Au-NPs.

#### Fluorescence Quenching by Ag-NPs Study

The fluorescence emission of Triazine I was studied in the presence of Ag-NPs in two solvents of different viscosities, namely ethanol and ethylene glycol. While a wide range of solvents was used to study the solvatochromic behavior of the dye alone, only ethanol and ethylene glycol were selected in the dye-nanoparticle interaction experiments to specifically investigate the influence of solvent viscosity on the interaction mechanism.

**Fig. 14:**
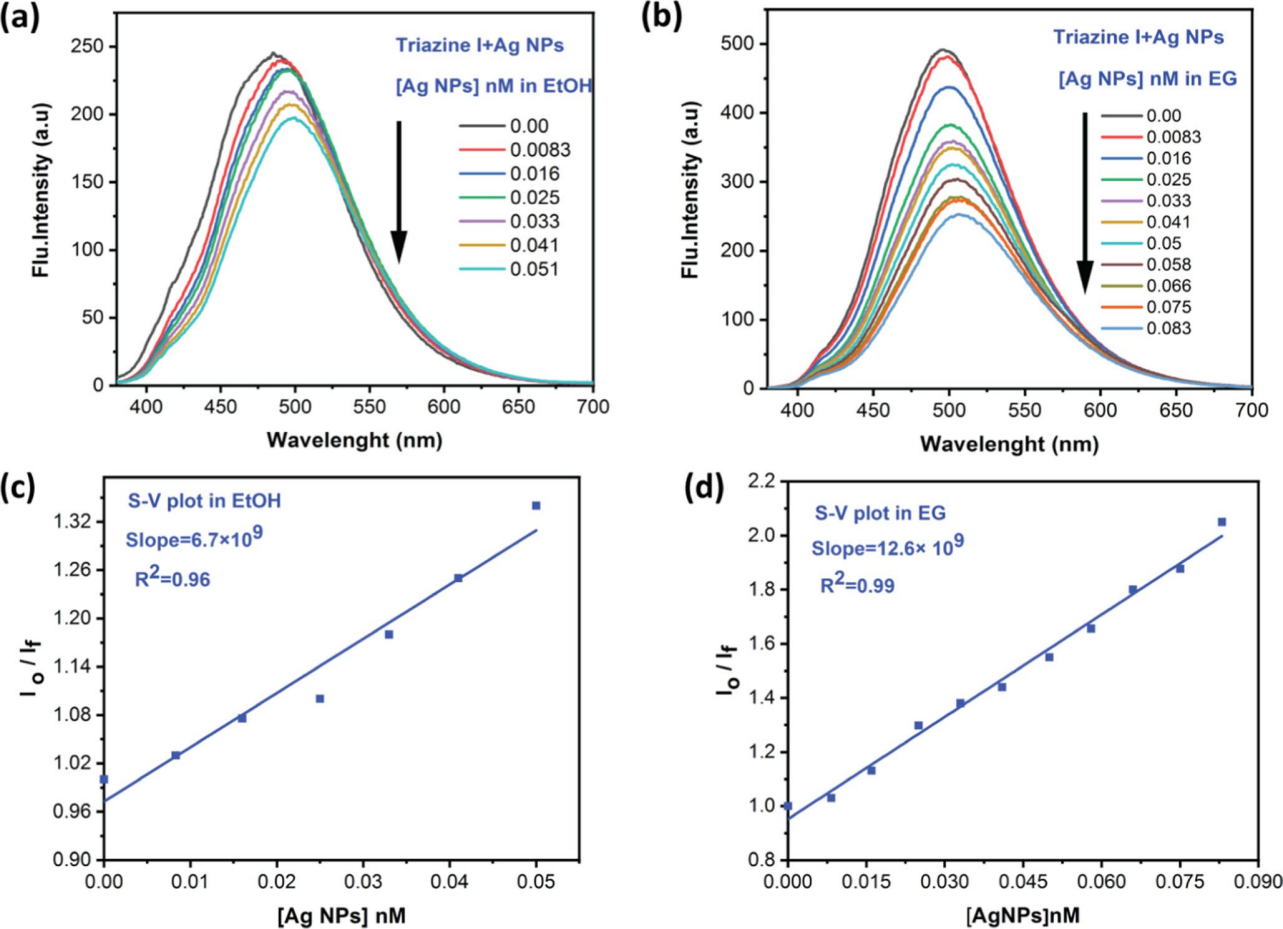
(**a** and **b**) Emission spectra of (1 × 10^−5^) mol L^−1^ of Triazine I in ethanol (EtOH) and ethylene glycol (EG) in the presence of different concentrations of Ag-NPs; (**c** and **d**) Linear Stern-Volmer plots for quenching Triazine I fluorescence using Ag-NPs

As shown in Fig. 14 (a and b), the emission intensity of Triazine I decreases as the Ag-NPs concentration increases with a slight red shift (~ 6 nm). This bathochromic shift can be attributed to changes in the microenvironment of the fluorophore due to ground-state complex formation with the quencher. Linear Stern-Volmer plots according to Eq. 11 [63] are given in Fig. 14 (c and d), both in ethanol and ethylene glycol, having correlation coefficients (R^2^) of 0.96 and 0.99, respectively.

11 $$\:\frac{{I}_{0}}{{I}_{f}}\:=\:1+{K}_{sv}\left[Q\right]\:\:\:\:\:\:\:\:\:\:\:\:\:\:$$

Where I_o_ and I_f_ are the fluorescence emission intensities in the absence and presence of the quencher concentration [Q], respectively. K_SV_ is the Stern-Volmer quenching constant (K_SV_ = k_q_.τ_f_), where k_q_ is the bimolecular quenching rate constant and τ_f_ is the excited state lifetime of Triazine I. The Stern-Volmer constants K_SV_ values were determined as 6.7 × 10^9^ M^−1^in ethanol and 12.6 × 10^9^ M^−1^ in ethylene glycol. The higher K_SV_ value in ethylene glycol suggests that quenching is not purely diffusion-controlled and that quenching efficiency rises with increasing medium viscosity.

This is associated with a static quenching concept where a cage effect caused by an increase in medium viscosity improves fluorophore uptake on Ag-NPs surfaces [64]. The calculated Stern–Volmer constants (K_SV_ values in the range of 10⁹ M⁻¹) are several orders of magnitude higher than typical static quenching values reported in the literature (~ 10²–10³ M⁻¹) [65–67], indicating unusually strong complex formation between the dye and Ag NPs. Notably, these values fall within the commonly accepted range for “super-quenching” systems (10⁷–10¹⁰ M⁻¹), confirming that the observed quenching behavior can be accurately described as superquenching consistent with literature definitions.

Taking the fluorescence lifetime of Triazine I in the absence of Ag-NPs as 2.1 ns, the values of k_q_ = K_sv_/τ_f_ are calculated as 3.19 × 10^18^ M^−1^ s^−1^ in ethanol and 6 × 10^18^ M^−1^s^−1^ in ethylene glycol. These values are about eight orders of magnitude higher than the diffusion rate constants k_d_ in both solvents (k_d_ = 9.2 × 10^10^ M^−1^ s^−1^) for ethanol and (k_d_ = 5.8 × 10^8^ M^−1^s^−1^) for ethylene glycol [68]. Based on this, it can be concluded that the mechanism of the static quenching and Forest-type energy transfer has a dominant role in Triazine I quenching by Ag-NPs.This was substantiated by measuring fluorescence decay curves of Triazine I as a function of Ag-NPs, as shown in Fig. S8, in which the excited state lifetime remains unchanged as the quencher [Ag-NPs] concentrations are changed.

The so-called hyper- or super-fluorescence quenching is responsible for the unusually high values of the fluorescence quenching rate constants [69]. Fluorescence super-quenching using metallic nanoparticles is generally caused by three factors: (1) a large absorption coefficient of the metallic nanoparticles’ surface plasmon resonance (SPR) absorption band; (2) the spherical shape of Ag-NPs, which permits energy transfer to occur at any orientation of the donor concerning the non metal surface; and (3) overlap between the donor’s emission and the SPR absorption band.

The volume of the quenching sphere radius (V in cm^3^) and the quenching sphere radius (r in nm) could be determined using the Perrin model for Triazine I quenching by Ag-NPs [70]. The Perrin relationship is given by Eq. (12)

12 $$\:\mathrm{ln}\left(\frac{{I}_{0}}{{I}_{f}}\right)=V{N}_{0}\left[Q\right]\:;\:\mathrm{V}=\:\frac{4}{3}\pi\:{r}^{3}$$

Where I_o_ and I_f_ represent the fluorescence emission intensities in the absence and presence of quencher, V represents the quenching sphere’s volume in cubic centimeters, N_o_ is Avogadro’s number, [Q] is the quencher’s molar concentration, and r is the quenching sphere’s radius in nm.

**Fig. 15:**
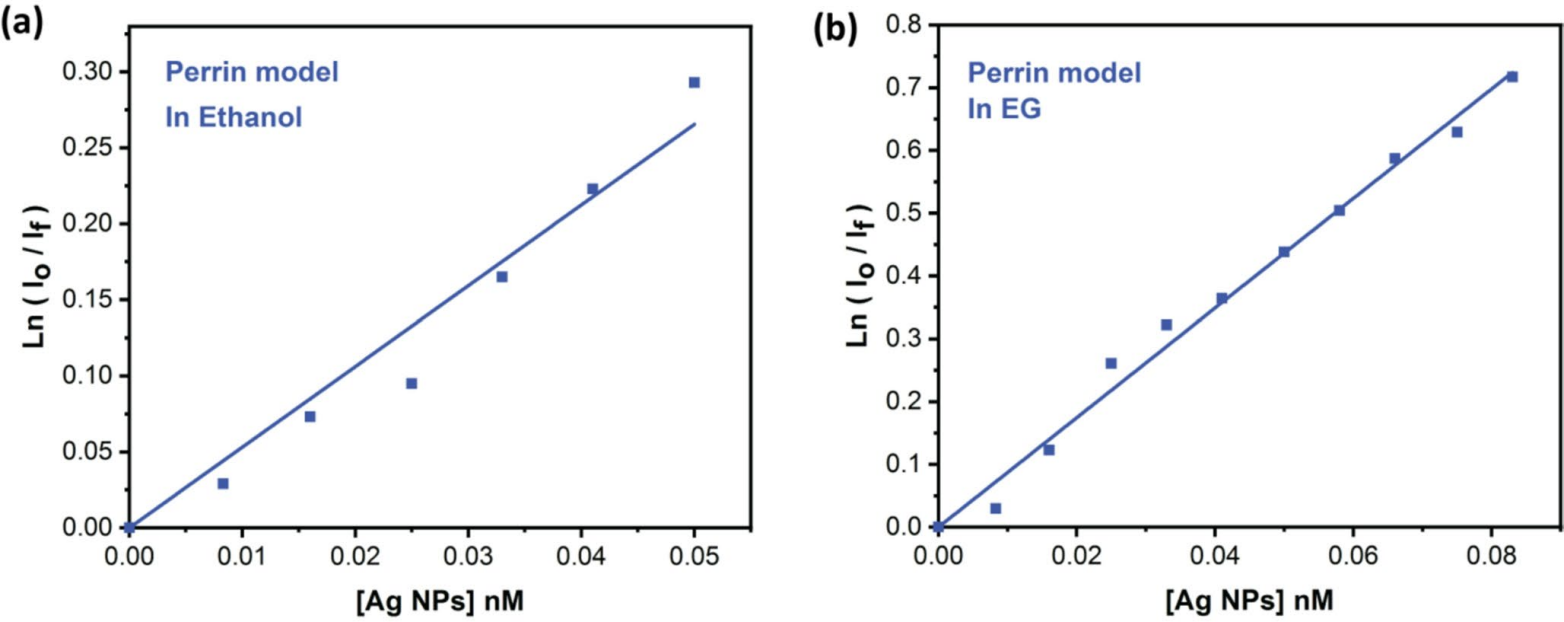
Perrin model for fluorescence super-quenching of Triazine I by Ag-NPs (**a**) in ethanol, (**b**) in EG

As shown in Fig. 15 (a and b), linear plots of ln(I_o_/I) versus [Q] with a slope equal to VN_o_.V and r values are calculated and listed in Table 4. The sum of molecular radii R = R_y_ + R_q_ is 25.41 nm, where R_y_ represents the fluorophore’s radius (0.41 nm) and R_q_ represents the quencher molecules’ radius (25 nm).

**Table 4:** Super-quenching photophysical parameters from Stern-Volmer plots (K_sv_ and K_q_ values) and the Perrin model (quenching sphere volume V, and radius r)

Solvent	Stern-volmer parameters		Perrin model parameters	
	K_sv_ (M^−1^)	K_q_ (M^−1^s^−1^)	V(cm^3^)	r (nm)
Ethanol	6.7 × 10^9^	3.19 × 10^18^	8.52 × 10^–15^	126
Ethylene glycol	12.6 × 10^9^	6 × 10^18^	1.39 × 10^–14^	149

The radius of the active sphere volume, as indicated in Table 4, was found to be larger than R, suggesting that radiative and non-radiative energy transfer processes are in charge of quenching the fluorescence of Triazine I. This is because, as shown in Fig. S9, there is a significant overlap between the Triazine I emission spectrum and the Ag-NPs plasmon surface, which improves the dipolar interaction between the excited state of Triazine I and the SPR nanoparticles.

#### Fluorescence Enhancement by Au-NPs

**Fig. 16:**
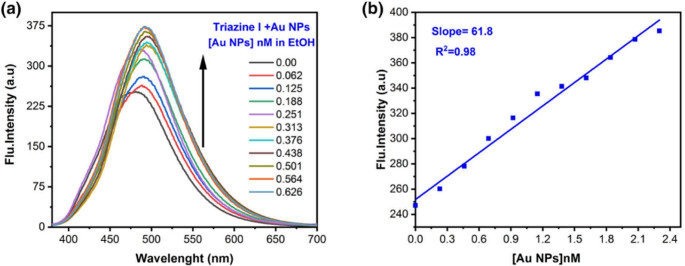
(**a**) Emission spectra of (1 × 10^−5^) mol L^−1^ of Triazine I in ethanol in the presence of different concentrations of Au-NPs; (**b**) a calibration curve for Au nanoparticles using constant concentrations of Triazine I (1 × 10^−5^) M and different concentrations of Au nanoparticles

The fluorescence spectra of (1 × 10^−5^) M of Triazine I in ethanol (λ_ex_. = 370 nm) were measured as a function of Au-NPs concentrations as shown in Fig. 16 (a). The fluorescence intensity of the dye is considerably enhanced upon increasing Au-NPs concentration, with a redshift from 480 nm to 492 nm.

**Fig. 17:**
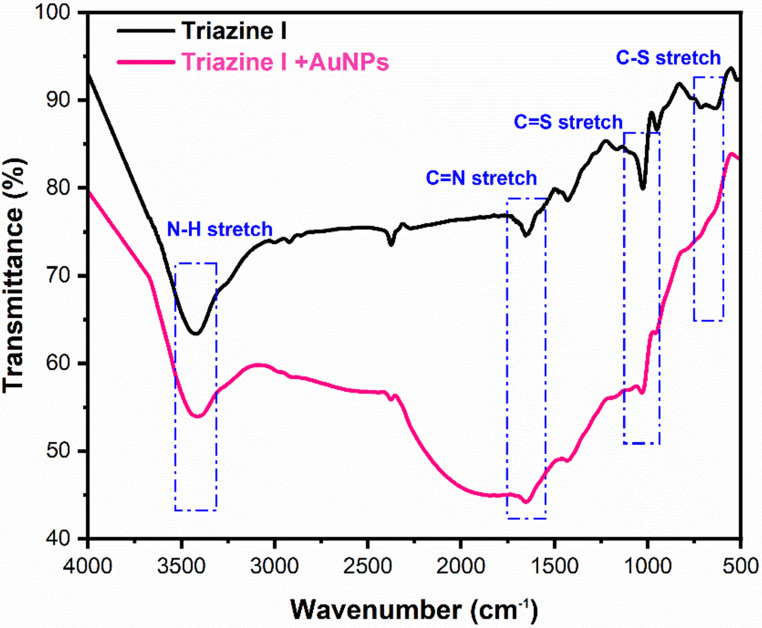
FTIR for Triazine I before and after the addition of Au-NPs

The dye mercapto group is known to have a high affinity to bind with Au-NPs via an S–Au bond [71]. This can be confirmed by using FTIR and Zeta potential. As shown in Fig. 17, after the addition of Au-NPs to Triazine I, noticeable changes appeared in the FTIR spectrum. The most significant change is the reduction or disappearance of the peak at ~ 1030 cm⁻¹, which is attributed to C = S stretching vibrations. This suggests a successful interaction between sulfur atoms in the dye and the gold surface, forming Au–S bonds.

Additionally, spectral shifts and intensity changes in the 626–700 cm⁻¹ region (assigned to C–S stretching) further support this interaction.

A broader spectral change was also observed between 1700 and 2200 cm⁻¹, which may be due to electronic environment alterations upon Au–S bonding, as well as plasmonic surface effects from the gold nanoparticles.

The zeta potential measurements provide clear evidence for sulfur-gold nanoparticle binding through systematic surface charge evolution as shown in Fig. S10. The gold nanoparticles alone exhibited a highly negative zeta potential (−31.03 mV), while the pure dye showed a moderate negative value (−10.50 mV). Upon conjugation, the resulting complex displayed an intermediate zeta potential (−15 mV), which represents a significant departure from simple additive behavior and indicates genuine chemical interaction. This shift, combined with the distinct changes in particle size distribution profiles observed in the power spectra (from a sharp, symmetric peak for pure dye to a broader, more complex distribution for the conjugate), conclusively demonstrates that sulfur atoms in the dye molecule have successfully coordinated with the gold nanoparticle surface, forming stable covalent bonds that modify the overall electrokinetic properties of the system. These results demonstrate strong binding of the dye to the Au-NP surface, which is a critical requirement for the MEF mechanism. The proximity enabled by this binding supports the observed enhancement and the 12 nm red shift in the emission spectra.

Gold nanoparticles exhibit fluorescence enhancement through a phenomenon called metal-enhanced fluorescence (MEF), initially detected in 2002 by Lakowicz and Geddes [72]. MEF has become the most sensitive technique for identifying a variety of analytes, including macromolecules, microorganisms, ions, and biomolecules.

In MEF, the electrons of the fluorophore move from the ground state (S_0_) to the excited singlet state (S_1_) when it is excited at a certain excitation wavelength. Before returning to the ground state, there are two methods by which excited electrons can lose energy: through radiative and non-radiative decays. The square of the electric field created by the interaction of the excitation light and surroundings is proportional to the rate of excitation. Once a fluorophore is placed close to a metal NP, the excitation and emission rates, radiation pattern, quantum yield, photostability, and radiative and non-radiative decay rates of the fluorophore will be modified. These alterations result from surface plasmon polaritons that are generated at the metal-dielectric interface when incident light with a wavelength that matches the metal plasmon resonance peak, which is usually intended to overlap with the fluorophore absorption and/or emission spectra, as shown in Fig. S11.

The increased electric field surrounding the metal nanoparticle causes an increase in the excitation rate. Because of the increased excitation rate, there will be a higher population of electrons in the excited state, which will accelerate both radiative and non-radiative decays. At the metal NP surface, the increased electric field reaches its maximum and is then exponentially reduced over long distances. Furthermore, another nonradiative process that decreases significantly when the distance between the fluorophore and nanoparticles increases is the energy transfer from the excited fluorophore to the metal NP. The spectrum overlap between the absorption of metal nanoparticles and fluorophore emission is a critical factor for creating ultrasensitive MEF biosensors that have significant enhancement parameters.

## Conclusions

In this study, we successfully synthesized and extensively characterized a new fused thieno-triazine derivative (Triazine I) using FTIR, ^1^H-NMR, ^13^C-NMR, and mass spectrometry to verify its structure and chemical composition. The optical and photophysical properties of Triazine I, including its significant intramolecular charge transfer (ICT) behavior and solvent sensitivity, were examined both experimentally and through DFT/TD-DFT calculations. Gold and silver nanoparticles (Au-NPs and Ag-NPs) were prepared by citrate reduction and comprehensively characterized using UV–vis spectroscopy (to identify surface plasmon resonance bands), TEM (for morphology), DLS (for size distribution), zeta potential measurements (for assessing colloidal stability), and XRD (to confirm crystalline structure). Importantly, this work reports for the first time metal-selective dual fluorescence modulation in a fused heterocyclic system. Triazine I exhibited distinct, metal-specific responses: fluorescence quenching with Ag-NPs via energy transfer and static quenching mechanisms, and significant fluorescence enhancement with Au-NPs through the metal-enhanced fluorescence (MEF) effect. This unprecedented dual behavior contrasts with typical systems showing only single-response modes. By integrating detailed experimental characterizations with theoretical modeling of electrostatic potential distributions, this study provides new mechanistic insights into how thieno fusion modulates photophysical properties and nanoparticle interactions. These findings establish a promising platform for designing highly selective and sensitive fluorescent probes for advanced optical sensing, bioimaging, and environmental monitoring applications.

## Electronic Supplementary Material

Below is the link to the electronic supplementary material.

Supplementary Material 1 (DOCX 1.79 MB)

## Data Availability

No datasets were generated or analysed during the current study.

## References

[1] Nugent BM, Buysse AM, Loso MR, Babcock JM, Johnson TC, Oliver MP, Martin TP, Ober MS, Breaux N, Robinson A (2015) Expanding the structure–activity relationship of sulfoxaflor: the synthesis and biological activity of N-heterocyclic sulfoximines. Pest Manag Sci 71(7):928–936. 10.1002/ps.3865 25067823 10.1002/ps.3865

[2] Kushwaha N, Sharma C (2020) The chemistry of triazine isomers: structures, reactions, synthesis and applications. Mini Rev Med Chem 20(20):2104–2122. 10.2174/1389557520666200729160720 32727324 10.2174/1389557520666200729160720

[3] Russomanno P, Assoni G, Amato J, D’Amore VM, Scaglia R, Brancaccio D, Pedrini M, Polcaro G, La Pietra V, Orlando P (2021) Interfering with the tumor–immune interface: making way for triazine-based small molecules as novel PD-L1 inhibitors. J Med Chem 64(21):16020–16045. 10.1021/acs.jmedchem.1c01409 34670084 10.1021/acs.jmedchem.1c01409

[4] Zhao S, Ali AS, Liu X, Yu Z, Kong X, Zhang Y, Savage GP, Xu Y, Lin B, Wu D (2024) 1, 3-Disubstituted-1, 2, 4-triazin-6-ones with potent activity against androgen receptor-dependent prostate cancer cells. Bioorg Med Chem 117634. 10.1016/j.bmc.2024.117634 10.1016/j.bmc.2024.117634 38359754

[5] Mushtaq A, Wu P, Naseer MM (2023) Recent drug design strategies and identification of key heterocyclic scaffolds for promising anticancer targets. Pharmacol Ther 108579. 10.1016/j.pharmthera.2023.108579 10.1016/j.pharmthera.2023.108579 38160914

[6] Sakr MA, Kana MTA (2022) 1, 2, 4-triazine-based materials: spectroscopic investigation, DFT, NBO, and TD-DFT calculations as well as dye-sensitized solar cells applications. J Fluoresc 32(6):2053–2063. 10.1007/s10895-022-03005-1 35861897 10.1007/s10895-022-03005-1 PMC9606079

[7] Fahim AM, Ismael EH, Elsayed GH, Farag AM, Ismael Eman H. I. (2022) Synthesis, antimicrobial, anti-proliferative activities, molecular docking and DFT studies of novel pyrazolo [5, 1-c][1, 2, 4] triazine-3-carboxamide derivatives. J Biomol Struct Dyn 40(19):9177–9193. 10.1080/07391102.2021.1930582 34106038 10.1080/07391102.2021.1930582

[8] Feng Y-Y, Dong C-E, Li R, Zhang X-Q, Wang W, Zhang X-R, Liu W-W, Shi D-H (2023) Design, synthesis and biological evaluation of quinoline-1, 2, 4-triazine hybrids as antimalarial agents. J Mol Struct 1271:133982. 10.1016/j.molstruc.2022.133982

[9] Mena L, Billamboz M, Charlet R, Desprès B, Sendid B, Ghinet A, Jawhara S (2022) Two new compounds containing pyridinone or triazine heterocycles have antifungal properties against *Candida albicans*. Antibiotics 11(1):72. 10.3390/antibiotics11010072 35052949 10.3390/antibiotics11010072 PMC8773291

[10] Li J, Zhang J (2022) The antibacterial activity of 1, 2, 3-triazole-and 1, 2, 4-triazole-containing hybrids against *Staphylococcus aureus*: an updated review (2020-present). Curr Top Med Chem 22(1):41–63. 10.2174/1568026621666211111160332 34766892 10.2174/1568026621666211111160332

[11] Gangasani JK, Yarasi S, Naidu VGM, Vaidya JR (2022) Triazine based chemical entities for anticancer activity. Phys Sci Rev 0. 10.1515/psr-2022-0005

[12] Bareth D, Jain S, Kumawat J, Kishore D, Dwivedi J, Hashmi SZ (2023) Synthetic and Pharmacological developments in the hybrid s-triazine moiety: A review. Bioorg Chem 106971. 10.1016/j.bioorg.2023.106971 10.1016/j.bioorg.2023.106971 38016395

[13] Marín-Ocampo L, Veloza LA, Abonia R, Sepúlveda-Arias JC (2019) Anti-inflammatory activity of triazine derivatives: a systematic review. Eur J Med Chem 162:435–447. 10.1016/j.ejmech.2018.11.027 30469039 10.1016/j.ejmech.2018.11.027

[14] Pal R, Kumar B, PM GS, Chawla PA (2023) Design, synthesis of 1, 2, 4-triazine derivatives as antidepressant and antioxidant agents: in vitro, in vivo and in Silico studies. Bioorg Chem 131:106284. 10.1016/j.bioorg.2022.106284 36444791 10.1016/j.bioorg.2022.106284

[15] Carbone D, De Franco M, Pecoraro C, Bassani D, Pavan M, Cascioferro S, Parrino B, Cirrincione G, Dall’Acqua S, Moro S (2023) Discovery of the 3-amino-1, 2, 4-triazine-based library as selective PDK1 inhibitors with therapeutic potential in highly aggressive pancreatic ductal adenocarcinoma. Int J Mol Sci 24(4):3679. 10.3390/ijms24043679 36835086 10.3390/ijms24043679 PMC9959349

[16] Xu J, Ma J, Peng Y, Cao S, Zhang S, Pang H (2023) Applications of metal nanoparticles/metal-organic frameworks composites in sensing field. Chin Chem Lett 34(4):107527. 10.1016/j.cclet.2022.05.041

[17] Montes-García V, Squillaci MA, Diez-Castellnou M, Ong QK, Stellacci F, Samori P (2021) Chemical sensing with Au and ag nanoparticles. Chem Soc Rev 50(2):1269–1304. 10.1039/D0CS01112F 33290474 10.1039/d0cs01112f

[18] Ghonim R, El-Awady MI, Tolba MM, Ibrahim FA (2023) Investigation of eco-friendly fluorescence quenching probes for assessment of Acemetacin using silver nanoparticles and acriflavine reagent. Sci Rep 13(1):4237. 10.1038/s41598-023-31106-9 36918612 10.1038/s41598-023-31106-9 PMC10014932

[19] Ghosh D, Chattopadhyay N (2015) Gold and silver nanoparticles based superquenching of fluorescence: a review. J Lumin 160:223–232. 10.1016/j.jlumin.2014.12.018

[20] Silvestre OF, Rao A, Liz-Marzán LM (2023) Self-assembled colloidal gold nanoparticles as substrates for plasmon enhanced fluorescence. Eur J Mater 3(1):2202676. 10.1080/26889277.2023.2202676

[21] Hamama WS, El-Bana GG, Shaaban S, Habib O, Zoorob HH (2016) Advances in the domain of 4-amino-3-mercapto-1, 2, 4-triazine-5-ones. RSC Adv 6(29):24010–24049. 10.1039/C5RA26433B

[22] Velapoldi R, Mielenz K (1981) A fluorescence standard reference material: quinine sulfate dihydrate. Appl Opt 20(9):1718–1718. 10.1364/AO.20.001718 20309374 10.1364/ao.20.001718

[23] Ullah S, Baloch MK, Nawaz M, Ur Rehman Z, Rehman W, Uddin I, Khan QU (2020) Investigation of stability and rheological properties of silver nanoparticles stabilized by polyethylene glycol. J Mater Sci Mater Electron 31:10470–10477. 10.1007/s10854-020-03595-1

[24] Hussain MH, Abu Bakar NF, Mustapa AN, Low K-F, Othman NH, Adam F (2020) Synthesis of various size gold nanoparticles by chemical reduction method with different solvent polarity. Nanoscale Res Lett 15:1–10. 10.1186/s11671-020-03370-5 32617698 10.1186/s11671-020-03370-5 PMC7332595

[25] De Souza CD, Nogueira BR, Rostelato MEC, Compounds (2019) Review of the methodologies used in the synthesis gold nanoparticles by chemical reduction. J Alloys 798:714–740. 10.1016/j.jallcom.2019.05.153

[26] Polte J, Erler R, Thunemann AF, Sokolov S, Ahner TT, Rademann K, Emmerling F, Kraehnert R (2010) Nucleation and growth of gold nanoparticles studied via in situ small angle X-ray scattering at millisecond time resolution. ACS Nano 4(2):1076–1082. 10.1021/nn901499c 20088602 10.1021/nn901499c

[27] Tofighi G, Lichtenberg H, Pesek J, Sheppard TL, Wang W, Schöttner L, Rinke G, Dittmeyer R, Grunwaldt J-D, Engineering (2017) Continuous microfluidic synthesis of colloidal ultrasmall gold nanoparticles: in situ study of the early reaction stages and application for catalysis. Reaction Chem 2(6):876–884. 10.1039/C7RE00114B

[28] Horiuchi T, Miura H, Sumioka K, Uchida S (2004) High efficiency of dye-sensitized solar cells based on metal-free Indoline dyes. J Am Chem Soc 126(39):12218–12219. 10.1021/ja0488277 15453726 10.1021/ja0488277

[29] Jia C, Wan Z, Zhang J, Li Z, Yao X, Shi Y (2012) Theoretical study of carbazole–triphenylamine-based dyes for dye-sensitized solar cells. Spectrochim Acta A Mol Biomol Spectrosc 86:387–391. 10.1016/j.saa.2011.10.053 22093522 10.1016/j.saa.2011.10.053

[30] AboAlhasan AA, Sakr MA, Abdelbar MF, El-Sheshtawy HS, El-Daly SA, Ebeid E-ZM, Al-Ashwal RH, Al-Hazmy SM (2022) Enhanced energy transfer from diolefinic laser dyes to meso-tetrakis (4-sulfonatophenyl) porphyrin immobilized on silver nanoparticles: DFT, TD-DFT and spectroscopic studies. J Saudi Chem Soc 26(4):101491. 10.1016/j.jscs.2022.101491

[31] Aboalhassan AA, El-Daly SA, Ebeid E-ZM, Sakr MA (2022) Plasmonic surface of metallic gold and silver nanoparticles induced fluorescence quenching of meso-terakis (4-sulfonatophenyl) porphyrin (TPPS) and theoretical–experimental comparable. J Fluoresc 32(6):2257–2269. 10.1007/s10895-022-03022-0 36045307 10.1007/s10895-022-03022-0 PMC9606071

[32] Sakr MA, Saad MA, Abdelsalam H, Abd-Elkader OH, Aleya L, Zhang Q (2023) Two-dimensional ZnS quantum dots for gas sensors: electronic and adsorption properties. J Electron Mater 52(8):5227–5238. 10.1007/s11664-023-10455-1

[33] Sakr ME, Abou Kana MT, Elwahy AH, El-Daly SA, Ebeid E-ZM (2020) Novel Far UV–Vis absorbing Bis (dihydrophenanthro [9, 10-e][1, 2, 4] triazine) derivative dyes: synthesis, optical, photophysical and solvatochromic properties. J Mol Struct 1206:127690. 10.1016/j.molstruc.2020.127690

[34] Sakr MA, Saad MA (2022) Spectroscopic investigation, DFT, NBO and TD-DFT calculation for porphyrin (PP) and porphyrin-based materials (PPBMs). J Mol Struct 1258:132699. 10.1016/j.molstruc.2022.132699

[35] Sakr MA, Mohamed AA, Abou Kana MT, Elwahy AH, El-Daly SA, Ebeid E-ZM (2021) Synthesis, characterization, DFT and TD-DFT study of novel Bis (5, 6-diphenyl-1, 2, 4-triazines). J Mol Struct 1226:129345. 10.1016/j.molstruc.2020.129345

[36] Aboalhassan AA, El-Daly SA, Ebeid E-ZM, Sakr MA (2022) 1, 4-bis [β-(2-benzoxazoly1) vinyl] benzene (BBVB) laser dye and sodium salt of meso-tetrakis (4-sulfonatophenyl) porphyrin (TPPS); spectroscopic investigation and DFT, NBO and TD-DFT calculations. J Photochem Photobiol A Chem 431:114039. 10.1016/j.jphotochem.2022.114039

[37] Sakr MA, Sherbiny FF, El-Etrawy A-AS (2022) Hydrazone-based materials; DFT, TD-DFT, NBO analysis, Fukui function, MESP analysis, and solar cell applications. J Fluoresc 32(5):1857–1871. 10.1007/s10895-022-03000-6 35737283 10.1007/s10895-022-03000-6 PMC9402755

[38] Sakr MA, El-Daly SA, Ebeid E-ZM, Al-Hazmy SM, Hassan M (2022) Quinoline-based materials: spectroscopic investigations as well as DFT and TD-DFT calculations. J Chem 2022:1–9. 10.1155/2022/1784406

[39] Frisch M, Trucks G, Schlegel H, Scuseria G, Robb M, Cheeseman J, Scalmani G, Barone V, Petersson G, Nakatsuji H (2016) Gaussian 16 revision C. 01, 2016. Gaussian Inc Wallingford CT 1:572

[40] El-Daly SA, Asiri AM, Alamry KA, Osman OI (2015) Synthesis, optical properties, laser activity and DFT studies of (E, E)-2, 5-bis [2-(1-methyl-1H-pyrrole-2-yl)-vinyl] pyrazine (BMPVP). J Photochem Photobiol A Chem 312:64–72. 10.1016/j.jphotochem.2015.07.003

[41] El-Daly SA, Alamry KA (2016) Spectroscopic investigation and photophysics of a D-π-A-π-D type styryl pyrazine derivative. J Fluoresc 26:163–176. 10.1007/s10895-015-1698-7 26498801 10.1007/s10895-015-1698-7

[42] Asiri AM, Alamry KA, Pannipara M, Al-Sehemi AG, El-Daly SA (2015) Spectroscopic investigation, photophysical parameters and DFT calculations of 4, 4′-(1E, 1′ E)-2, 2′-(pyrazine-2, 5-diyl) bis (ethene-2, 1-diyl) bis (N, N-dimethylaniline)(PENDA) in different solvents. Spectrochim Acta A Mol Biomol Spectrosc 149:722–730. 10.1016/j.saa.2015.05.018 25988818 10.1016/j.saa.2015.05.018

[43] Lu T, Chen F (2012) Multiwfn: a multifunctional wavefunction analyzer. J Comput Chem 33(5):580–592. 10.1002/jcc.22885 22162017 10.1002/jcc.22885

[44] Abdelsalam H, Saroka VA, Younis WO (2019) Phosphorene quantum dot electronic properties and gas sensing. Phys E Low-dimens Syst Nanostruct 107:105–109. 10.1016/j.physe.2018.11.012

[45] Aravindan P, Sivaraj K, Kamal C, Vennila P, Venkatesh G (2021) Synthesis, molecular structure, spectral characterization, molecular docking and biological activities of (E)-N-(2-methoxy benzylidene) anthracene-2-amine and Co (II), Cu (II) and Zn (II) complexes. J Mol Struct 1229:129488. 10.1016/j.molstruc.2020.129488

[46] Jana S, Dalapati S, Ghosh S, Guchhait N (2013) Excited state intramolecular charge transfer process in 5-(4-dimethylamino-phenyl)-penta-2, 4-dienoic acid ethyl ester and effect of acceptor functional groups. J Photochem Photobiol A Chem 261:31–40. 10.1016/j.jphotochem.2013.04.005

[47] Singh H, Sindhu J, Khurana JM (2014) Determination of dipole moment, solvatochromic studies and application as turn off fluorescence chemosensor of new 3-(4-(dimethylamino) phenyl)-1-(5-methyl-1-(naphthalen-1-yl)-1H-1, 2, 3-triazol-4-yl) prop-2-en-1-one. Sens Actuators B: Chem 192:536–542. 10.1016/j.snb.2013.10.137

[48] Tewari N, Joshi N, Rautela R, Gahlaut R, Joshi H, Pant S (2011) On the ground and excited state dipole moments of Dansylamide from solvatochromic shifts of absorption and fluorescence spectra. J Mol Liq 160(3):150–153. 10.1016/j.molliq.2011.03.008

[49] Lippert E (1955) Dipolmoment und elektronenstruktur von Angeregten Molekülen. Z Für Naturforschung A 10(7):541–545. 10.1515/zna-1955-0707

[50] Mataga N, Kaifu Y, Koizumi M (1956) Solvent effects upon fluorescence spectra and the dipolemoments of excited molecules. Bull Chem Soc Jpn 29(4):465–470. 10.1246/bcsj.29.465

[51] Suppan P (1983) Excited-state dipole moments from absorption/fluorescence solvatochromic ratios. Chem Phys Lett 94(3):272–275. 10.1016/0009-2614(83)87086-9

[52] Ravi M, Soujanya T, Samanta A, Radhakrishnan T (1995) Excited-state dipole moments of some coumarin dyes from a solvatochromic method using the solvent polarity parameter, ENT. J Chem Soc Faraday Trans 91(17):2739–2742. 10.1039/FT9959102739

[53] Reichardt C (2007) Solvents and solvent effects: an introduction. Org Process Res Dev 11(1):105–113. 10.1021/op0680082

[54] Patil S, Wari M, Panicker CY, Inamdar S (2014) Determination of ground and excited state dipole moments of dipolar laser dyes by solvatochromic shift method. Spectrochim Acta A Mol Biomol Spectrosc 123:117–126. 10.1016/j.saa.2013.12.031 24394528 10.1016/j.saa.2013.12.031

[55] Coe BJ, Harris JA, Asselberghs I, Clays K, Olbrechts G, Persoons A, Hupp JT, Johnson RC, Coles SJ, Hursthouse MB (2002) Quadratic nonlinear optical properties of N-Aryl stilbazolium dyes. Adv Funct Mater 12(2):110–116. 10.1002/1616-3028(20020201)12:2%3C110::AID-ADFM110%3E3.0.CO;2-Y

[56] Calzaferri G, Rytz RJ (1995) Electronic transition oscillator strength by the extended Hueckel molecular orbital method. J Phys Chem 99(32):12141–12150. 10.1021/j100032a015

[57] Lippert E (1970) Photophysical primary steps in solutions of aromatic compounds. Acc Chem Res 3(2):74–80. 10.1021/ar50026a006

[58] Shaikh M, Mohanty J, Singh P, Bhasikuttan A, Rajule R, Satam V, Bendre S, Kanetkar V, Pal H (2010) Contrasting solvent polarity effect on the photophysical properties of two newly synthesized aminostyryl dyes in the lower and in the higher solvent polarity regions. J Phys Chem A 114(13):4507–4519. 10.1021/jp9107969 20170148 10.1021/jp9107969

[59] Shim T, Lee MH, Kim D, Ouchi Y (2008) Comparison of photophysical properties of the hemicyanine dyes in ionic and nonionic solvents. J Phys Chem B 112(7):1906–1912. 10.1021/jp076757v 18220383 10.1021/jp076757v

[60] Mourdikoudis S, Pallares RM, Thanh NT (2018) Characterization techniques for nanoparticles: comparison and complementarity upon studying nanoparticle properties. Nanoscale Res Lett 10(27):12871–12934. 10.1039/C8NR02278J 10.1039/c8nr02278j 29926865

[61] Biju V, Itoh T, Anas A, Sujith A, Ishikawa M (2008) Semiconductor quantum dots and metal nanoparticles: syntheses, optical properties, and biological applications. Anal Bioanal Chem 391:2469–2495. 10.1007/s00216-008-2185-7 18548237 10.1007/s00216-008-2185-7

[62] Lin P-C, Lin S, Wang PC, Sridhar R (2014) Techniques for physicochemical characterization of nanomaterials. Biotechnol Adv 32(4):711–726. 10.1016/j.biotechadv.2013.11.006 24252561 10.1016/j.biotechadv.2013.11.006 PMC4024087

[63] Juneau A, Hope TO, Malenfant J, Mesko M, McNeill J, Frenette M (2022) Methods to predict potential reagents in iridium-based photoredox catalysis calibrated with stern–volmer quenching rate constants. ACS Catal 12(4):2348–2356. 10.1021/acscatal.1c04740

[64] El-Daly SA, Salem IA, Hussein MA, Asiri AM (2015) Fluorescence quenching N, N-bis (2, 6-dimethylphenyl)-3, 4: 9, 10-perylenetetracarboxylic diimide (BDPD) laser dye by colloidal silver nanoparticles. J Fluoresc 25:379–385. 10.1007/s10895-015-1523-3 25656068 10.1007/s10895-015-1523-3

[65] Lakowicz JR, Weber G (1973) Quenching of fluorescence by oxygen. Probe for structural fluctuations in macromolecules. Biochemistry 12(21):4161–4170. 10.1021/bi00745a020 4795686 10.1021/bi00745a020 PMC6959846

[66] Eftink MR, Ghiron CA (1981) Fluorescence quenching studies with proteins. Anal Biochem 114(2):199–227. 10.1016/0003-2697(81)90474-7 7030122 10.1016/0003-2697(81)90474-7

[67] Lehrer S (1971) Solute perturbation of protein fluorescence. Quenching of the tryptophyl fluorescence of model compounds and of lysozyme by iodide ion. Biochemistry 10(17):3254–3263. 10.1021/bi00793a015 5119250 10.1021/bi00793a015

[68] El-Daly SA, Rahman MM, Alamry KA, Asiri AM (2014) Fluorescence quenching of N, N-bis (2, 5-di-tert-butylphenyl)-3, 4: 9, 10-perylenebis (dicarboximide)(DBPI) by silver nanoparticles. J Lumin 148:303–306. 10.1016/j.jlumin.2013.12.026 10.1007/s10895-014-1415-y 24903127

[69] Morozov VN, Kolyvanova MA, Dement’eva OV, Rudoy VM, Kuzmin VA (2020) Fluorescence superquenching of SYBR green I in crowded DNA by gold nanoparticles. J Lumin 219:116898. 10.1016/j.jlumin.2019.116898

[70] Wang X, Bai Y, He Q, Li J, Wang S, Guo W, Sun X (2024) Preparation and p-phenylenediamine detection mechanism of a dialdehyde cellulose and a 7-amino-4-methylcoumarin-based fluorescent probe. Int J Biol Macromol 254:127783. 10.1016/j.ijbiomac.2023.127783 37924904 10.1016/j.ijbiomac.2023.127783

[71] Liu P, Qin R, Fu G, Zheng N (2017) Surface coordination chemistry of metal nanomaterials. J Am Chem Soc 139(6):2122–2131. 10.1021/jacs.6b10978 28085260 10.1021/jacs.6b10978

[72] Geddes CD, Lakowicz J (2002) Metal-enhanced fluorescence. J Fluoresc 12:121–129. 10.1023/A:1016875709579

