# Supplementary material for: Optical and Computational Studies on a Triazine Derivative of Dual Fluorescence Enhancement/Superquenching Upon Nanoparticle Interactions
Source: J Fluoresc. 2025 Sep 25;36(1):11–31. doi: 10.1007/s10895-025-04474-w (PMC12957092; doi:10.1007/s10895-025-04474-w)
Supplement: Supplementary file 1 — Supplementary Material 1 (DOCX 1.79 MB) [file 10895_2025_4474_MOESM1_ESM.docx]

**Supporting information for**

**Optical and Computational Studies on a Triazine Derivative of Dual Fluorescence Enhancement / Superquenching Upon Nanoparticle Interactions.**

Khloud M. Elhalby^a,*^, Ahmed H. Mangood^a^, Mahmoud A. S. Sakr^b^, Ahmed A. El-Barbary^c^, El-Zeiny M. Ebeid^c^, and Heba A. El-Shekheby^a, *^

^a^Chemistry Department, Faculty of Science, Menoufia University, Shebin El-Kom, Egypt.

^b^Chemistry Department, Center of Basic Science, Misr University for Science and Technology (MUST), P.O.77, Giza, Egypt.

^c^Chemistry Department, Faculty of Science, Tanta University, Tanta 31527, Egypt.

^*^Corresponding author: Khloud M. Elhalby, [khloudelhalby@gmail.com](mailto:khloudelhalby@gmail.com)

**Synthesis of Triazine I dye**

4-amino-6-(4-methoxyphenyl)-1,4,6,7-tetrahydrothieno[2,3-e] [1,2,4] triazine-3(2H)-thione (Triazine I) was synthesized via a two-step process that involves the sequential condensation of thiocarbohydrazide with arylidenepyruvic acid, followed by cyclization using phosphorus pentasulfide. **In the first one**, Thiocarbohydrazide (1.062 g, 10.0 mmol, 1.0 equiv.) compound (1) was condensed with 4-methoxycinnamylpyruvic acid (2.730 g, 11.0 mmol, 1.1 equiv.) compound (2) in anhydrous dioxane (25 mL) at 100°C for 5 hours under a nitrogen atmosphere. The reaction proceeds via nucleophilic attack of the thiocarbohydrazide nitrogen on the carbonyl carbon, followed by imine formation and subsequent cyclization to yield 6-(arylvinyl)-4-amino-3-mercapto-1,2,4-triazin-5(2H)-one (1.89 g, 7.2 mmol) compound (3) in 72% yield after recrystallization from ethanol (30 mL). **In the second step**, the intermediate compound (3) from Step 1 (1.89 g, 7.2 mmol, 1.0 equiv.) was treated with phosphorus pentasulfide (P₂S₅, 0.960 g, 4.32 mmol, 0.6 equiv.) in anhydrous pyridine (20 mL) at 110°C for 4 hours to yield compound (4). P₂S₅ serves as a sulfurizing agent, promoting intramolecular cyclization through dehydration and ring closure, while pyridine functions as both solvent and base, neutralizing HCl generated during the reaction. The reaction mixture was cooled and poured into ice water (100 mL), and the precipitated product was filtered and purified by recrystallization from DMF (15 mL) to afford Triazine I. The synthetic route is outlined in Scheme 1, and the structure of the compound was confirmed by FT-IR spectra (Fig. S1), EI-mass (Fig. S2), ^1^H-NMR (Fig. S3), and ^13^C-NMR spectra (Fig. S4). The physical and spectral criteria of Triazine I are: Yield 78%, reddish orange crystals, m.p.: 312-315 ^o^C; (EI) m/z: calcd for [C_12_H_12_N_4_OS_2_] 292.37; found, 292.38; IR(KBr): ν = 1631 (CS), 1598 (CN), 2959 (CH), 3241 (NH_2_) cm^-1^; ^1^HNMR (DMSO-d_6_): δ (ppm) 3.53 (s, 3H, OCH₃), 3.81 (s, 3H, OCH₃), 4.25 (br s, 2H, NH₂), 6.49 (s, 1H, Ar–H), 6.94 (d, J = 7.8 Hz, 1H, Ar–H), 7.05 (d, J = 8.4 Hz, 1H, Ar–H), 7.58 (d, J = 8.7 Hz, 1H, Ar–H), 7.75 (s, 1H, Ar–H), 7.80 (d, J =8.1 Hz, 1H, Ar–H); 13CNMR: δ (ppm) =160.2 (C=S), 158.4 (C-OCH₃), 128.99 (Ar-C), 115.44 (Ar-C), 100.0 (Ar-C), 55.3 (OCH₃), 40.41 (CH₂); C_12_H_12_N_4_OS_2_ (292.38); Calcd: C, 49.30; H, 4.14, N, 19.16. Found: C, 49.14; H, 4.11; N, 19.08. This synthetic approach creates a unique fused thieno-triazine system with distinct electronic properties responsible for the observed dual fluorescence modulation behavior.





**Scheme 1** Preparation of (Triazine I; compound 4) (Ar = MeOC_6_H_5_).





**Fig. S1** FTIR spectra for Triazine I





**Fig. S2** EI-MS for Triazine I


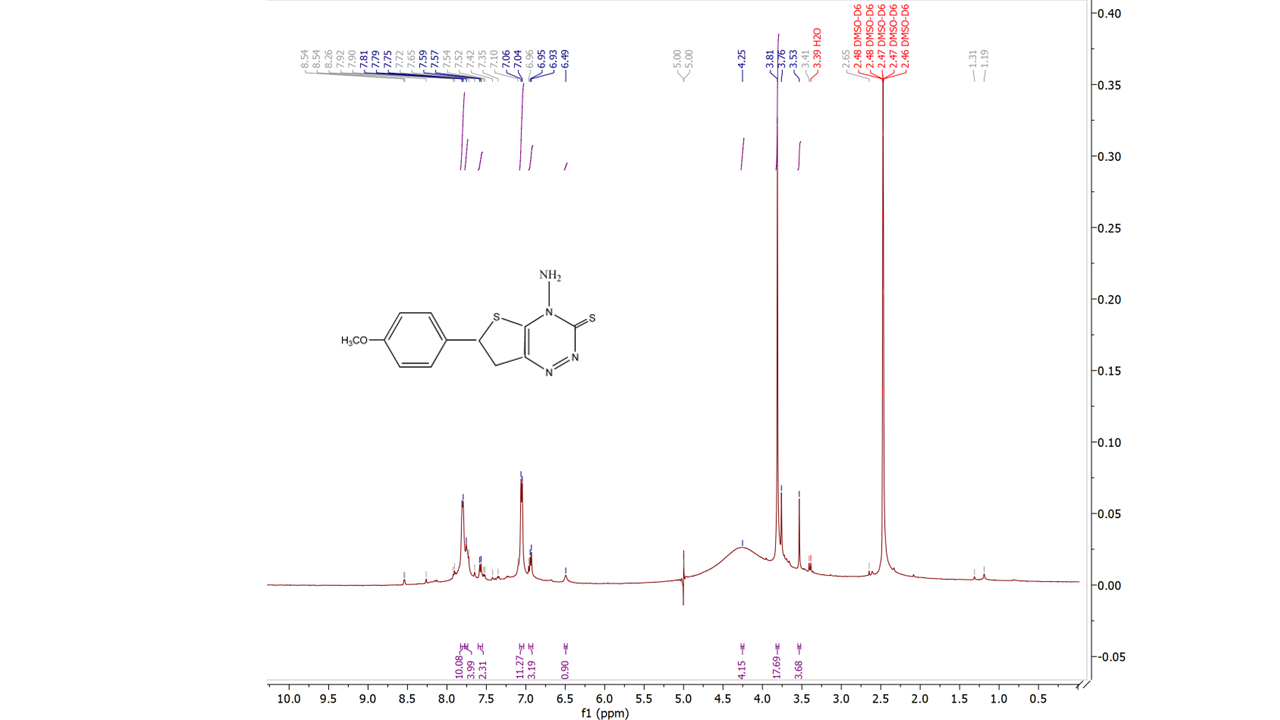


**Fig. S3** ^1^H-NMR of Triazine I in DMSO-d_6_.


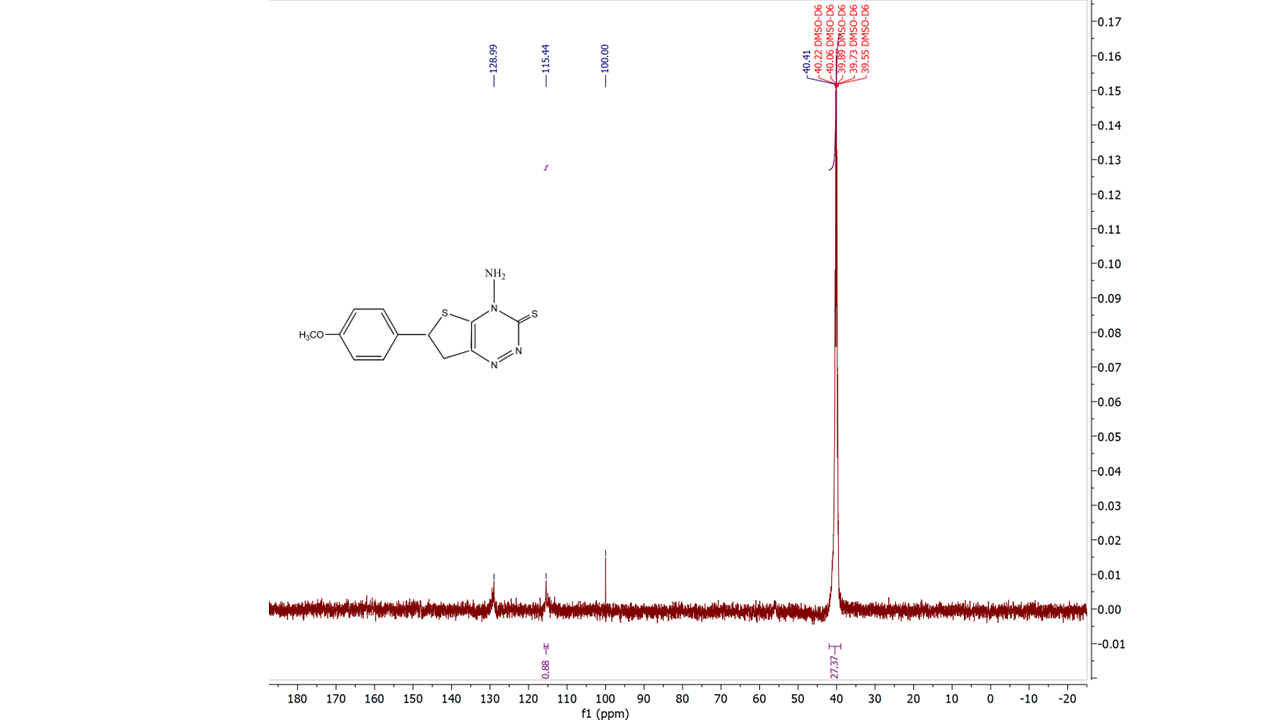


**Fig. S4** ^13^C-NMR of Triazine I in DMSO-*d_6_*.


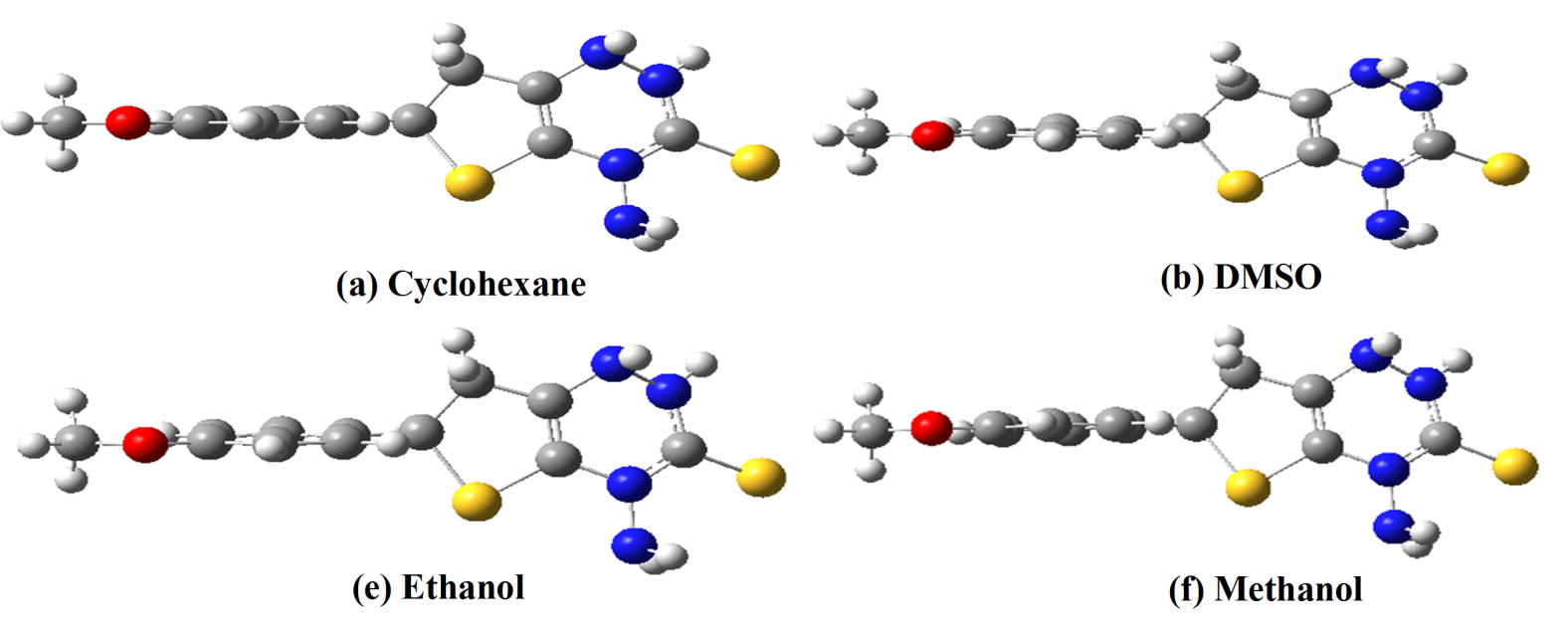


**Fig. S5** Optimized structures of triazine I in different solvents.


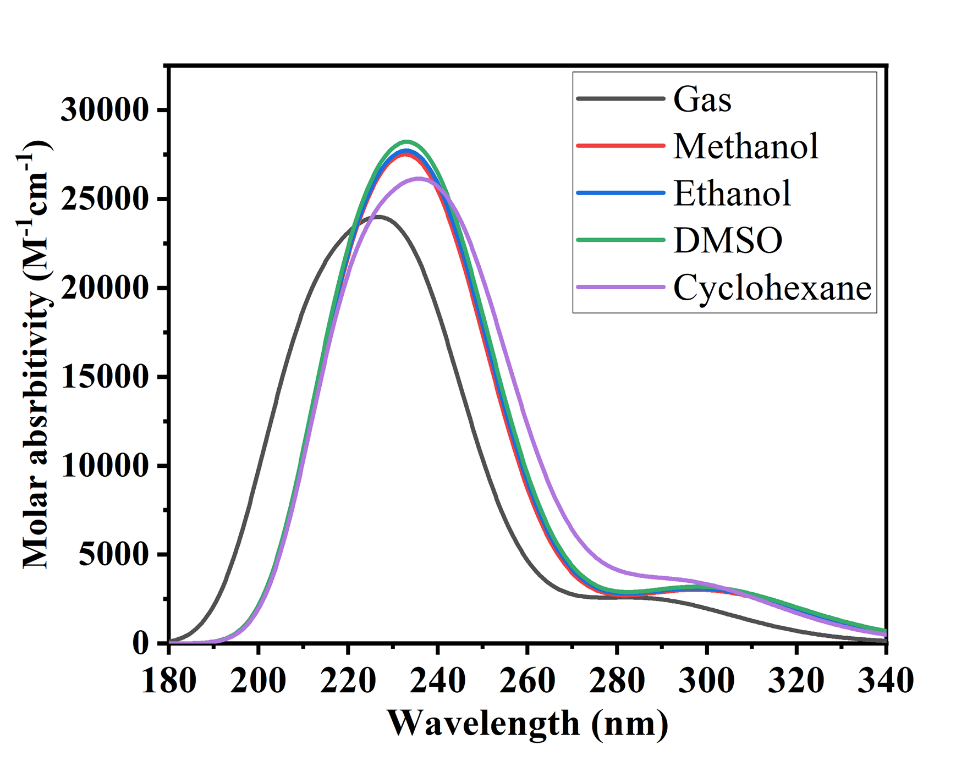


**Fig. S6** The computational electronic absorption spectra for Triazine I in various solvents.





**Fig. S7** Fluorescence quantum yields of Triazine I versus Microscopic Polarity parameter of the solvent $E_{T}^{N}$ in varying solvents: 1) cyclohexane, 2) Dioxane, 3) THF, 4) DMF, 5) DMSO, 6) Propanol, 7) Ethanol, 8) Methanol.





**Fig. S8** Excited state lifetime decay curves of (1×10^-5^) M ethanolic Triazine I solutions as a function of [Ag-NPs] quencher concentration (λ_ex._ = 370 nm).





**Fig. S9** Spectral overlap of (a) silver nanoparticles' absorption spectra with (b) Triazine I's emission spectrum.


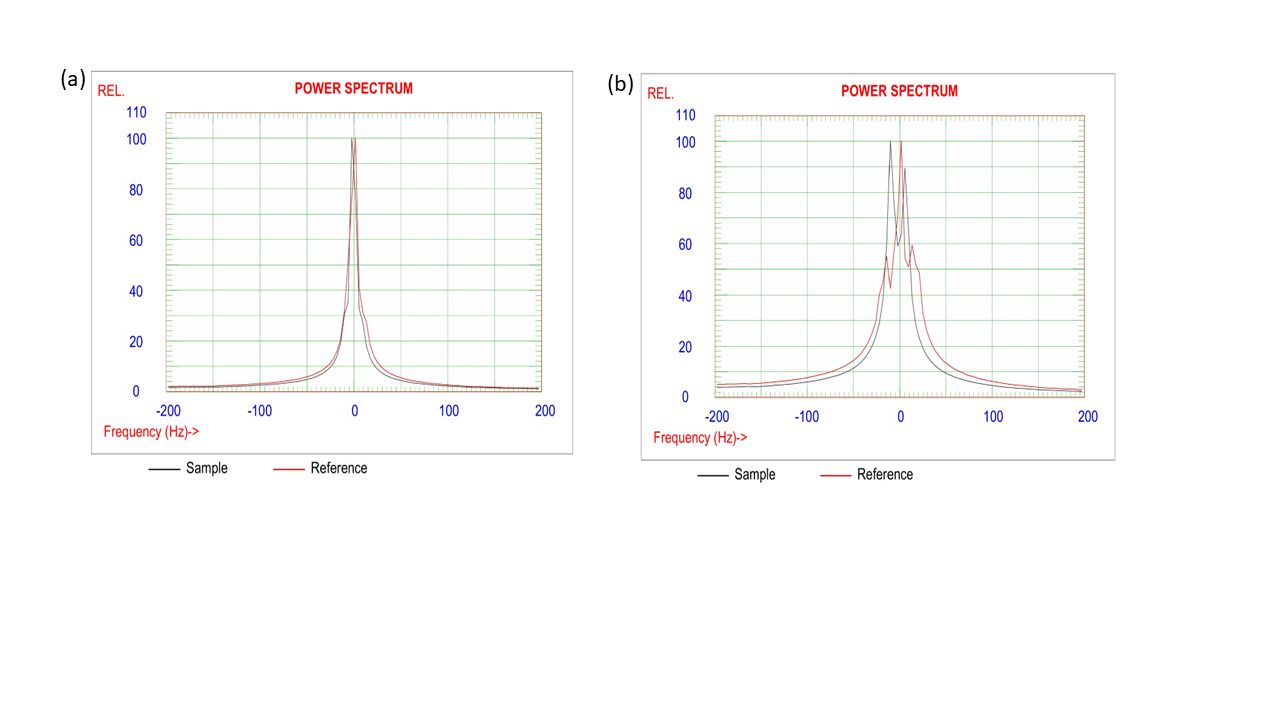


**Fig. S10** Zeta potential for (a) Triazine I and (b) Triazine I+ Au-NPs.





**Fig. S11** An overlap of (a) the emission spectra of Triazine I with (b) the absorption spectra of gold nanoparticles.
